# Classification of phytoplankton cells as live or dead using the vital stains fluorescein diacetate and 5‐chloromethylfluorescein diacetate

**DOI:** 10.1111/jpy.12415

**Published:** 2016-04-28

**Authors:** Hugh L. MacIntyre, John J. Cullen

**Affiliations:** ^1^ Department of OceanographyDalhousie UniversityPO Box 15000HalifaxNova ScotiaB3H 4R2Canada

**Keywords:** confidence intervals, false negative, false positive, flow cytometry, mortality, most probable number, viability, vital stain, vitality

## Abstract

Regulations for ballast water treatment specify limits on the concentrations of living cells in discharge water. The vital stains fluorescein diacetate (FDA) and 5‐chloromethylfluorescein diacetate (CMFDA) in combination have been recommended for use in verification of ballast water treatment technology. We tested the effectiveness of FDA and CMFDA, singly and in combination, in discriminating between living and heat‐killed populations of 24 species of phytoplankton from seven divisions, verifying with quantitative growth assays that uniformly live and dead populations were compared. The diagnostic signal, per‐cell fluorescence intensity, was measured by flow cytometry and alternate discriminatory thresholds were defined statistically from the frequency distributions of the dead or living cells. Species were clustered by staining patterns: for four species, the staining of live versus dead cells was distinct, and live‐dead classification was essentially error free. But overlap between the frequency distributions of living and heat‐killed cells in the other taxa led to unavoidable errors, well in excess of 20% in many. In 4 very weakly staining taxa, the mean fluorescence intensity in the heat‐killed cells was higher than that of the living cells, which is inconsistent with the assumptions of the method. Applying the criteria of ≤5% false negative plus ≤5% false positive errors, and no significant loss of cells due to staining, FDA and FDA+CMFDA gave acceptably accurate results for only 8–10 of 24 species (i.e., 33%–42%). CMFDA was the least effective stain and its addition to FDA did not improve the performance of FDA alone.

**Abbreviations**
ACD: average cell diameter
CCMP: Strain designation for phytoplankton cultures from National Center for Marine Algae and Microbiota
CMFDA: 5‐chloromethylfluorescein diacetate
ETV: Environmental Technology Verification
FDA: fluorescein diacetate
HDPE: high‐density polyethylene
LLD: lower limit of detection
UTEX: The Culture Collection of Algae at the University of Texas at Austin

The movement of goods by sea is a fundamental feature of the global economy. The simultaneous transport of live organisms in the ships' ballast water is one means by which cyanobacteria and microalgae can be introduced to new environments. These include taxa that have the potential to cause ecological and economic damages (e.g., Ruiz et al. 2000, Doblin et al. 2004, Roy et al. 2012). In response to the threat of invasive species, mandatory treatment of ballast water has been proposed by the UN International Maritime Organization (IMO 2004) and has been codified in law by the United States Congress in the *Nonindigenous Aquatic Nuisance Prevention and Control Act* (1990) and the *National Invasive Species Act* (1996). In both regulatory regimes, the concentrations of potentially invasive organisms in ballast water must meet discharge standards. The IMO (2004) expresses these in terms of “viable” cells whereas the USA regulations (DHS 2012) specify “living” cells. However, for the purpose of their approval guidelines, the IMO (2008) defines “viable” as “living.”

The boundary between life and death in phytoplankton and bacteria is not clear and there is no widely agreed definition of what delineates one from the other (reviewed by Franklin et al. 2006, Davey 2011, Berges and Choi 2014). However, recognizing that the distinction between “viable” and “living” can be critically important in the evaluation of ballast water management systems (First and Drake 2013, Cullen and MacIntyre 2016), we define our terms specifically as they apply to microorganisms such as phytoplankton subjected to binary classification as live versus dead.

Viability is the ability to increase in cellular biomass and reproduce. This process requires three conditions: an intact cytoplasmic membrane; DNA transcription and RNA translation; and generation of energy for anabolic metabolism (Breeuwer and Abee 2000; Fig. 1). As has long been recognized (e.g., Redford and Myers 1951), reproductive potential and metabolic competence are not synonymous: cells may exhibit signs of metabolic competence such as photosynthesis but have a severely reduced capacity to reproduce (i.e., be vital but not viable). Conversely, resting stages such as cysts may show no sign of vitality but be viable on excystment. Consequently, we reserve use of the term viability to denote the capacity for cell proliferation, and vitality to denote demonstration of one or more of the three metabolic prerequisites (intact membranes, nucleotide functionality, and/or metabolic competence) defined by Breeuwer and Abee (2000). Here, the terms living and live refer to cells that are vital; in turn, live versus dead is a binary classification of cells as living or not living.

**Figure 1:**
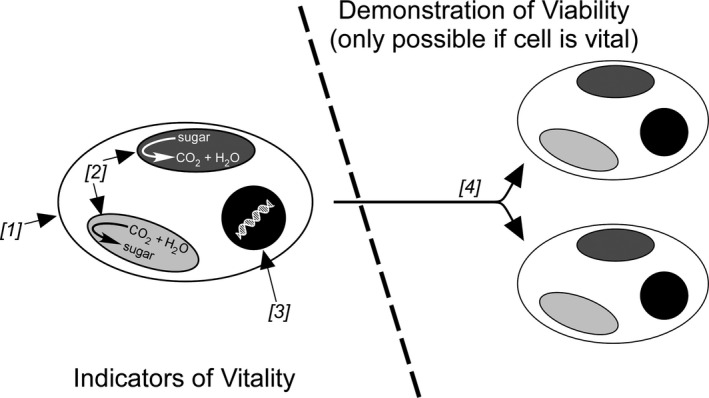
Tests of vitality and viability, after Breeuwer and Abee (2000). Tests that confirm the integrity of the cell membrane (1), the ability of the cell to generate energy for biosynthetic reactions through respiration or photosynthesis (2), or that demonstrate DNA transcription and RNA translation (3) are indicators of vitality, through which a cell can be classified as living. Observation of cell growth and proliferation (4) is a demonstration of viability and is only possible if the cell has an intact membrane, is metabolically competent, and has functional nucleotides (indicators 1–3). A viable cell is therefore vital. However, a cell can test positively for one of indicators 1–3 but lack another prerequisite for reproduction and so be classified as vital although not viable.

Regulatory oversight of ballast water treatment in the USA has been assigned to the United States Coast Guard, which has adopted tests that were recommended in the Environmental Technology Verification (ETV) protocol (EPA 2010) to discriminate between live and dead cells for regulatory compliance (DHS 2012). These are based on two fluorescent “vital” stains, fluorescein diacetate and 5‐chloromethylfluorescein diacetate (FDA and CMFDA). The former is a fluorophore quenched by two acetyl groups that can easily diffuse through the cell membrane. Cleavage of the acetyl groups by esterases inside a cell with an intact membrane leads to accumulation of the product, a fluorophore that is less membrane‐permeable (Rotman and Papermaster 1966). Staining with CMFDA is also based on easy passage of the quenched substrate and retention of the fluorescent product in cells with intact membranes, but the target is different: CMFDA is thiol‐reactive (Poot et al. 1991), losing the quenching acetyl groups by thiol‐aided hydrolysis. For both stains, an assessment of vitality can be made by comparing the fluorescence of stained cells with a suitable control, such as a heat‐treated sample that is considered to be uniformly dead (Steinberg et al. 2011).

From its earliest applications with phytoplankton, it has been evident that FDA is not unambiguously reliable as an indicator of vitality. In addition to interspecific variability of per‐cell staining intensity (Dorsey et al. 1989, Agustí and Sanchez 2002, Garvey et al. 2007, Reavie et al. 2010, Peperzak and Brussaard 2011), there can also be intraspecific variability that reflects the cells' environmental history. Cells may show staining in normal media but not when exposed to high Cu^2+^ concentrations even though still swimming (i.e., obviously living; Bentley‐Mowat 1982); heterogeneity of staining can be high in some actively growing cultures in which all cells should be robust (Selvin et al. 1988); cultures undergoing significant cell loss, likely through programmed cell death, may stain uniformly and strongly (Selvin et al. 1988); staining intensity may differ between exponentially growing cells and cells in pre‐exponential phase (Gilbert et al. 1992) or nutrient‐stressed cells in stationary phase (Brookes et al. 2000, Garvey et al. 2007), and also between cells grown at different irradiances (Brookes et al. 2000); and staining intensity may show a pronounced diel pattern (Gilbert et al. 1992). Green autofluorescence produces a confounding signal in certain taxa (Tang and Dobbs 2007) and, where fluorescence is evaluated by epifluorescence microscopy rather than flow cytometry, the detection of green fluorescence from the stain can be compromised by the red fluorescence of chl *a* (Garvey et al. 2007) and relatively high levels of background green fluorescence from esterases released by lysed cells (Agustí et al. 1998).

Because of the numerous sources of variability in vital stain fluorescence intensity and the continuum between clearly living and clearly dead cells (Franklin et al. 2006, Davey 2011, Berges and Choi 2014), it is not surprising that conspecific individuals sampled from natural populations can have staining intensities that indicate a gradient rather than a strict binary division suited to easy classification of cells as live versus dead (e.g., fig. 2 in Fux et al. 2010, fig. 2, t and y in Reavie et al. 2010, Wright and Welschmeyer 2015). Frequency distributions of staining intensity in unialgal cultures, measured with flow cytometry, also show high intraspecific variability in cells with identical life histories, and the frequency distributions of live and dead cells can overlap (Dorsey et al. 1989, Lage et al. 2001, Debenest et al. 2011, Peperzak and Brussaard 2011). However, for the assessment of ballast water management systems using vital stains, the assessment is binary: only live cells are counted against the regulatory standard. Any overlap in the frequency distributions introduces a corresponding uncertainty into the assessment because any cell with a staining intensity in the overlapping region of the distributions might be alive or might be dead. This would necessitate the use of one or more complementary tests to augment the staining. Detection of movement has been used to identify cells that have been incorrectly classified by stains as dead (e.g., Steinberg et al. 2011, Adams et al. 2014), but this is also subject to uncertainty: many phytoplankton such as centric diatoms are not motile, some dinoflagellates lose mobility after exposure to FDA (Selvin et al. 1988), a significant proportion of actively‐growing flagellates may be immobile during microscopic examination (e.g., c. 5‐30% in Sheng et al. 2010), and flagellated cells can lose motility rapidly in the bright illumination of a microscope stage (Knight‐Jones 1951).

Various other assessments of vitality or mortality (i.e., the absence of vitality), including cell digestion assays, nucleic acid stains, and nonfluorescing vital stains, have been tested with phytoplankton, but so far, none appear to be more reliable than FDA (Reynolds et al. 1978, Veldhuis et al. 1997, Reavie et al. 2010, Peperzak and Brussaard 2011, Zetsche and Meysman 2012, Imase et al. 2013). Such comparisons are difficult to evaluate, though, because the accuracy of FDA and CMFDA as a binary measure of cell vitality has yet to be assessed quantitatively and consistently for a broad range of phytoplankton taxa.

The purpose of this study was to determine the accuracy of FDA and CMFDA, alone and in combination, in classifying individual phytoplankton cells as live or dead. Using quantitative measures of cellular fluorescence intensity from actively growing and heat‐killed cultures in quantitative, replicated and rigorously controlled experiments, we have determined the accuracy of these vital stains with 24 species of phytoplankton. Staining with FDA and CMFDA was assessed with flow cytometry and compared to results for the combined stain, FDA+CMFDA, that was recommended by the ETV panel (EPA 2010). We found that each of the three stain assays failed to discriminate between live and dead cells with greater than 90% confidence in the majority of the species tested. Epifluorescence microscopy, the method recommended by the ETV protocol for discriminating live from dead cells based on staining intensity, was evaluated by estimating the range of fluorescence intensity thresholds that an operator would have to apply when discriminating between live and dead cells in a mixed population. We conclude that it is very wide and likely to be a significant impediment to accurate assessment. Although this study is focused on two vital stains, its results are relevant to the quantitative assessment of other cell signals that can be measured with flow cytometry for application to a binary classification.

## Materials and Methods

### Distinguishing live from dead cells based on a diagnostic signal

We consider the classification of vitality from the perspective of the U. S. ballast water regulations, which stipulate that the concentrations of living cells in discharge must not exceed a specified threshold (DHS 2012). Accurate determination of the concentration of live cells is the sole objective of regulatory testing, and with vital stains the classification must be based on a threshold for the diagnostic signal. If the signal associated with vitality is normally distributed in both live and dead populations, the ability to discriminate them can be expressed in terms of an overlapping coefficient based on statistical estimates of those distributions (Inman and Bradley 1989). When the samples are large enough to describe the probability density functions explicitly rather than with mean and standard deviation, as is the case when flow cytometry is used to measure the diagnostic signal in thousands of cells, the overlap between populations, and thus the rates of error in classification, can be calculated directly.

**Figure 2:**
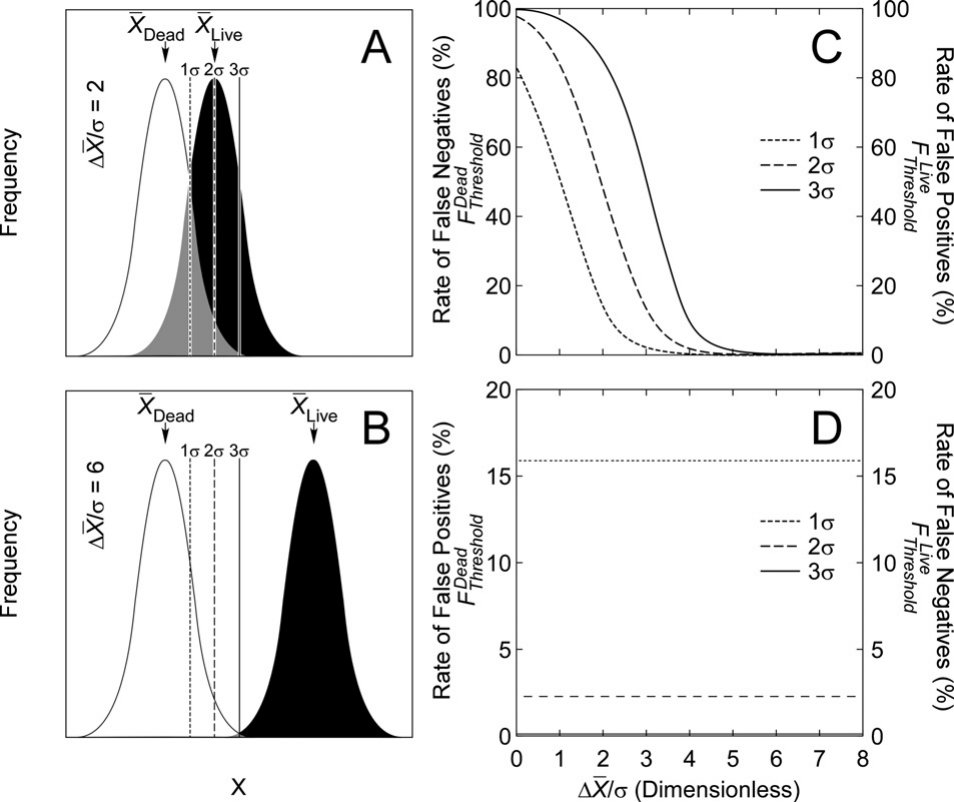
(A, B) Frequency distributions of a diagnostic variable *X* for hypothetical dead and live populations with the means, ${\overset{¯}{X}}_{Dead}$ and ${\overset{¯}{X}}_{Live}$. Both distributions have the same standard deviation, σ. The degree of separation is parameterized as $\Delta \overset{¯}{X}/\sigma$. The gray area is the overlap between the distributions. Discriminatory thresholds of 1, 2, and 3 σ above the lower mean, ${\overset{¯}{X}}_{Dead}$, are shown as vertical lines. (C) Rate of false negatives when classification is based on the statistically‐not‐dead approach ($X_{Threshold}^{Dead}={\overset{¯}{X}}_{Dead}+T\sigma$) as a function of population separation for each of the three classificatory thresholds, *T* = 1, 2, and 3 σ above ${\overset{¯}{X}}_{Dead}$. False negatives are live individuals that are misclassified as dead because the measurement is less than $X_{Threshold}^{Dead}$. If the classification is based on statistically‐not‐alive approach ($X_{Threshold}^{Live}={\overset{¯}{X}}_{Live}-T\sigma$), this graph shows the rate of false positives. False positives are dead individuals misclassified as live because the measurement is greater than $X_{Threshold}^{Live}$. (D) Statistically fixed rate of false positives (statistically‐not‐dead approach) or false negatives (statistically‐not‐alive approach) as a function of population separation for each of the three classificatory thresholds described for [C]. The line corresponding to 3σ is at 0.14%.

Consider the frequency distributions of a diagnostic variable such as staining intensity in two populations of phytoplankton that are unambiguously dead and alive (Fig. 2). If there is an overlap in the frequency distributions of two populations, the use of a single threshold value to define a boundary between them gives rise to two types of misclassification. False negatives are cells that have been classified as dead that are in fact alive. As discussed by Cullen and MacIntyre (2016) in the context of compliance with ballast water regulations, false negatives are potential propagules that are undetected. These may lead to regulatory acceptance of systems that exceed discharge limits for living cells, rendering the regulations less protective of the environment than intended. False positives are nonvital cells that are erroneously counted as live: in effect, they make the regulations more stringent than specified by discharge standards. These errors are conservative with respect to regulation because risk to the environment is reduced (DHS 2012, as discussed by Cullen and MacIntyre 2016), but manufacturers or ship operators could be unfairly penalized if compliant ballast water management systems failed to meet the standard due to erroneous test results. Both types of errors reduce the accuracy of ballast water testing, but false negatives are of particular concern because systematic errors of this type correspond to increased risk to the environment.

The rate of false negatives is $n_{<Th}^{Live}/n_{Total}^{Live}$, where $n_{<Th}^{Live}$ is the number of live cells with a signal lower than the threshold and $n_{Total}^{Live}$ is the total number of live cells. The rate of false positives is $n_{>Th}^{Dead}/n_{Ttoal}^{Dead}$, where $n_{>Th}^{Dead}$ is the number of dead cells with a signal higher than the threshold and $n_{Total}^{Dead}$ is the total number of dead cells. For two normally distributed populations with equal variances, the rates of both false negative and false positive errors can be related to the means and standard deviations of the two populations and the stringency of the thresholds. The values can be calculated from the areas of the normal distribution above and below a given value, using tabulated coefficients (Sokal and Rohlf 1981). Consider a live/dead classification based on the frequency distribution of the diagnostic signal from dead cells, *X*_*Dead*_. A threshold can be set to the mean (${\overset{¯}{X}}_{Dead}$) plus some multiple *T* of the standard deviation, σ_*Dead*_, i.e.,:

(1) $$X_{Threshold}^{Dead}={\overset{¯}{X}}_{Dead}+T\cdot \sigma_{Dead}$$

Cells with a signal at or below the threshold ($X\leq X_{Threshold}^{Dead}$) are classified as statistically indistinguishable from dead (Fig. 2, A and B) and correspondingly those above it are classified as live. We refer to this as the “statistically‐not‐dead” classification. False negatives result when the signal from live cells is less than the threshold value, as is common when frequency distributions of the live and dead populations overlap (Fig. 2A compared to 2B). The proportion increases when the threshold is raised to enhance statistical confidence in the “not dead” classification (e.g., increasing *T* so that $X_{Threshold}^{Dead}={\overset{¯}{X}}_{Dead}+3\sigma_{Dead}$ vs. $X_{Threshold}^{Dead}=\overset{¯}{X}+1\sigma_{Dead}$, Fig. 2, A and C). The influence of overlap on the rate of error is conveniently expressed as a function of the dimensionless ratio $\Delta \overset{¯}{X}/\sigma =({\overset{¯}{X}}_{Live}-{\overset{¯}{X}}_{Dead})/\sigma$, where ${\overset{¯}{X}}_{Live}$ and ${\overset{¯}{X}}_{Dead}$ are mean signals for the normal distributions of live and dead cells and σ is the standard deviation, here assumed to be the same for each (Fig. 2C). False negative errors are vanishingly small when $\Delta \overset{¯}{X}/\sigma \geq 6$ (Fig. 2C). Because the statistically‐not‐dead threshold is based solely on the frequency distribution of signal from dead cells, the rate of false positive results is statistically fixed (Fig. 2D) and has nothing to do with the signal from live cells.

For the vital stains considered here, the diagnostic signal is *F*_*green*_, green fluorescence per cell. Consistent with the fact that signals from FDA and CMFDA are used to identify live cells, the statistically‐not‐dead approach considers cells to be dead by default and then, by analogy with an analytical lower limit of detection (LLD; Anderson 1989, Miller and Miller 2005), defines an upper limit of the distribution of the dead cells' fluorescence intensities. This is the threshold for classification of a cell as live, $F_{Threshold}^{Dead}$. A commonly used criterion in analytical chemistry (Miller and Miller 2005) defines the LLD as 3 standard deviations above the mean of the blank. By analogy, $F_{Threshold}^{Dead}$ would be the lowest per‐cell fluorescence that a living cell could have and still be discriminated from dead cells with statistical confidence. Given that the cytometric frequency distributions had to be log‐transformed to approximate normality (see Results), we defined the threshold as:

(2) $$\text{log}\left(F_{Threshold}^{Dead}\right)=\bar{\text{log}(F_{Dead})}+3{\text{SD}}_{Dead}$$

where $\bar{\text{log}(F_{Dead})}$ is the mean log‐transformed fluorescence intensity of the dead cells and SD_*Dead*_ is the standard deviation for the log distribution. Cells with fluorescence higher than $F_{Threshold}^{Dead}$ are classified as live and those with fluorescence equal to or lower than $F_{Threshold}^{Dead}$ are classified as dead.

Using the frequency distribution of the dead cells to define the classificatory threshold (eq. (1)) minimizes false positives at the expense of increasing the likelihood of false negatives (Fig. 2, C and D). This type of error can result in weakened environmental protection. In principle, the distribution of signals from living cells could be used as a baseline for detecting the absence of vitality. In this alternative “statistically‐not‐alive” classification, the threshold would be defined as:

(3) $$\text{log}\left(F_{Threshold}^{Live}\right)=\bar{\text{log}(F_{Live})}-3{\text{SD}}_{Live}$$

where the notation is parallel to equation (2), except that the cut‐off is defined with reference to the distributions of the living cells: cells with signals below the threshold are classified as dead and those equal to or above it are classified as live. The difference is that this classification minimizes false negatives at the expense of increasing the likelihood of false positives (Fig. 2, C and D).

An alternative, nonparametric approach can be used when the observed populations are large enough to approximate accurately the frequency distributions of the diagnostic signal. Its primary advantage is that it does not rely on parameterized descriptions of either the living or dead populations. In the statistically‐not‐dead approach, if $F_{Threshold}^{Dead}$ is defined as the 95th percentile of the distribution of signal from the dead cells, then the rate of false positives is fixed at 5%, but potentially at the expense of false negatives. Conversely, in the statistically‐not‐alive approach, if $F_{Threshold}^{Live}$ is defined as the 5th percentile of the distribution of signal from the living cells, the rate of false negatives is 5% and the rate of false positives will increase if there is significant overlap in the distributions.

### Culture maintenance

Cultures of 24 species of marine and freshwater phytoplankton from seven divisions (Table 1) were obtained from the National Center for Marine Algae and Microbiota (East Boothbay, ME, USA) and from The Culture Collection of Algae at the University of Texas at Austin (Austin, TX, USA). The cultures were grown on f/2 (Guillard 1975) or L1 (Guillard and Hargraves 1993) seawater media or on Fritz freshwater f/2 medium (Fritz Industries, Dallas, TX, USA), as described in Table 1. Seawater growth media were prepared from tangential‐flow‐filtered coastal seawater (salinity ~30) that was enriched with nutrients and delivered into autoclaved glassware through a 0.2‐μm Whatman Polycap AS disposable filter capsule. Freshwater medium was prepared from E‐Pure water (Barnstead Nanopure/APS Water Services Corporation, Lake Balboa, CA, USA). All glassware and tubing with which the cultures made contact was cleaned by soaking in Micro‐90 cleaning solution (Z281506; Sigma‐Aldrich), rinsed copiously with E‐Pure water, soaked in 10% HCl, rinsed again with E‐Pure water, and dried in an inverted position before being sterilized in an autoclave. All transfers were done in a laminar flow hood with 0.2‐μm filtered air and the tubes were flamed on opening and closing to minimize the risk of contamination.

**Table 1:** Cultures used in the study. Strain numbers are from the National Center for Marine Algae and Microbiota (CCMP) or The Culture Collection of Algae at the University of Texas at Austin (UTEX). Cultures were grown on f/2 or L1 (seawater) or Fritz (freshwater) media, as indicated. Specific growth rate, μ_F_, is calculated from the between‐day values (mean ± SD, 100 < *n* < 450). ACD is average cell diameter, calculated from cross‐sectional area (mean ± SD, *n* > 50)

Division	Species	Growth medium	μ_F_ (d^−1^)	ACD (μm)
Cyanophyta	*Synechococcus elongatus* Nägeli (CCMP1630)	f/2	0.63 ± 0.06	N.D.
Chlorophyta	*Chlamydomonas* cf. sp. (CCMP1268)	f/2	0.40 ± 0.09	11.7 ± 1.6
	*Dunaliella tertiolecta* Butcher (CCMP1320)	f/2	0.60 ± 0.06	9.0 ± 0.9
	*Micractinium* sp. (UTEXLB2614)	Fritz	0.79 ± 0.06	7.1 ± 1.2
	*Nephroselmis pyriformis* (Carter) Ettl (CCMP717)	L1	0.83 ± 0.08	4.2 ± 0.5
	*Pyramimonas parkeae* Norris et Pearson (CCMP725)	L1	0.58 ± 0.05	13.1 ± 1.5
	*Scenedesmus obliquus* (Turpin) Kützing (UTEX393)	Fritz	0.75 ± 0.05	7.0 ± 1.3
Euglenophyta	*Eutreptiella* cf. *gymnastica* (CCMP1594)	L1	0.41 ± 0.07	23.7 ± 4.3
Cryptophyta	*Rhodomonas salina* (Wislouch) Hill et Wetherbee (CCMP1319)	L1	0.64 ± 0.08	11.6 ± 1.6
	*Storeatula* sp. (CCMP1868)	L1	0.42 ± 0.07	15.3 ± 1.7
Haptophyta	*Chrysochromulina kappa* Parke et Manton (CCMP288)	L1	0.57 ± 0.06	6.0 ± 0.7
	*Isochrysis galbana* Parke (CCMP1323)	L1	0.67 ± 0.04	5.2 ± 0.6
	*Phaeocystis globosa* Scherffel (CCMP627)	L1	0.81 ± 0.08	7.6 ± 1.1
	*Prymnesium parvum* Carter (CCMP1926)	L1^a^	0.65 ± 0.08	7.0 ± 1.2
Heterokontophyta	*Amphiprora* sp. (CCMP467)	f/2	0.80 ± 0.08	14.4 ± 1.1
	*Chaetoceros simplex* Ostenfeld (CCMP200)^b^	f/2	0.89 ± 0.08	5.1 ± 0.9^c^
	*Heterosigma akashiwo* (Y.Hada) Y.Hada ex Y.Hara & M.Chihara (CCMP1912)	L1	0.56 ± 0.07	15.5 ± 2.8
	*Thalassiosira pseudonana* (Hustedt) Hasle et Heimdal (CCMP1335)	f/2	1.06 ± 0.10	6.0 ± 0.4
	*Thalassiosira weissflogii* (Grunow) Fryxell et Hasle (CCMP1050)	f/2	0.83 ± 0.05	13.0 ± 1.4
Dinophyta	*Alexandrium andersoni* Balech (CCMP3376)	L1	0.31 ± 0.05	22.8 ± 2.4
	*Amphidinium carterae* Hulbert (CCMP1314)	f/2	0.53 ± 0.06	13.5 ± 1.5
	*Heterocapsa* sp. (CCMP451)	L1	0.30 ± 0.07	15.5 ± 2.8
	*Prorocentrum triestinum* Schiller (CCMP1919)	L1	0.42 ± 0.07	17.0 ± 1.9
	*Scrippsiella trochoidea* (Stein) Loeblich III (CCMP2775)	L1	0.41 ± 0.08	20.4 ± 3.7

a Enriched on transfer with 150 μM NH_4_Cl.

b Note that *C. simplex* is a unicellular, not chain‐forming, strain.

c Measurement does not include setae.

The cultures were maintained in semicontinuous growth (MacIntyre and Cullen 2005) in 50‐mL Pyrex tubes closed with unlined high‐density polyethylene (HDPE) caps. The growth incubator was maintained at 18°C ± 0.5°C with illumination of 80 μmol photons · m^−2^ · s^−1^ of PAR supplied by Osram FL40SS‐W/37 fluorescent bulbs on a 12/12 light/dark cycle. The position of tubes on the incubator shelves was defined to ensure consistent illumination within 5% of the nominal irradiance, as measured with a QSL‐101 quantum scalar irradiance detector (Biospherical Instruments, San Diego, CA, USA) in fluid‐filled tubes.

Chl *a* fluorescence of the cultures was monitored daily, 3–5 h into the dark period, by inserting the 50‐mL tubes into a Turner 10AU fluorometer (Turner Designs, Sunnyvale, CA, USA) and a FIRe benchtop fluorometer (Satlantic, Halifax, NS, Canada). Blanks (filtered seawater) and standards were measured daily. A solid standard supplied by the manufacturer was used with the 10AU and the FIRe was standardized with a 100 μmol · L^−1^ solution of rhodamine *b* (R6625; Sigma‐Aldrich, Oakville, ON, Canada) in E‐Pure water. The minimum, maximum, and variable fluorescence (*F*_0_, *F*_*m*_, *F*_*v*_) and the dimensionless ratio *F*_*v*_/*F*_*m*_ were obtained by nonlinear fitting of the fluorescence induction curve using Fireworx software (Audrey Barnett, http://sourceforge.net/projects/fireworx/). Curve fits were performed with the curvature parameter in the fit, *p*, set to zero.

A daily specific growth rate, μ_F_, (d^−1^) was calculated from the between‐day increase in dark‐acclimated fluorescence (Parkhill et al. 2001, Wood et al. 2005):

(4) $$\mu_{F}=\frac{1}{t_{2}-t_{1}}\cdot \ln\left(\frac{F_{2}\cdot {\left[1-d\right]}^{-1}}{F_{1}}\right)$$

where *t*_1_ and *t*_2_ are the times (*d*) of successive observations, *F*_1_ and *F*_2_ (Arb.) are the corresponding blank‐corrected fluorescence obtained from the Turner 10AU, and *d* (dimensionless) is the dilution (proportion of culture replaced with fresh medium) between readings.

The cultures were diluted every 1–5 generations with fresh medium to maintain them in exponential growth at low optical density. The cultures were assumed to be in balanced growth and ready for experimental use when either: (i) the coefficients of variation in μ_F_ and *F*_*v*_/*F*_*m*_ were less than 10% over 10 generations; or (ii) when the coefficient of determination of a linear regression of *ln*‐transformed, dilution‐corrected fluorescence on time was >0.995 and the coefficient of variation for *F*_*v*_/*F*_*m*_ was <10% over 10 generations. The former criteria were used for rapidly growing cultures (μ_F_ ≥0.5 d^−1^; Table 1) and the latter were used for slower‐growing cultures (μ_F_ <0.5 d^−1^; Table 1), for which the signal:noise on estimates of daily fluorescence increases effectively precluded accurate daily estimation of μ_F_ by equation (4).

### Harvesting and heat treatment

Cells were harvested 4–6 h after the start of the dark period when the conditions for balanced growth had been met: 1 mL aliquots were dispensed into screw‐capped HDPE cryovials for staining (see below) without further treatment or after being heated. These are referred to as “untreated” and “heat‐treated” below. The protocol for the latter followed Steinberg et al. (2011): the cryovials were immersed in a water bath at 50°C for 10 min but re‐equilibrated to the temperature in the growth chamber (18°C ± 0.5°C) for 20 min prior to staining.

### Staining

Cultures were stained with two vital stains, FDA and CMFDA (Life Technologies, Burlington, ON, Canada), both singly and in combination. The procedures followed protocols described by Steinberg et al. (2011). A primary stock of FDA in reagent‐grade acetone (12 mmol · L^−1^) was diluted with chilled E‐Pure water to prepare a secondary stock (480 μmol · L^−1^) immediately before analysis. The secondary stock was held on ice and used within 2 h of preparation. A primary stock of CMFDA in reagent‐grade dimethyl sulfoxide (250 μmol · L^−1^) was used without further dilution. The secondary FDA and primary CMFDA stocks were added to 1‐mL aliquots of culture for final concentrations of 5 and 2.5 μmol · L^−1^, respectively. The samples were incubated in darkness at 18°C for 10 min before measuring fluorescence intensity. Cultures were stained in a 2 × 4 matrix of untreated and heat‐treated cells that were each examined without staining and after staining with FDA alone, CMFDA alone, and FDA and CMFDA in combination. At least three independent cultures of each taxon were tested for staining (see Results).

The frequency distributions of per‐cell stain intensity were measured as green fluorescence, *F*_*green*_, with an Accuri C6 flow cytometer (Becton Dickinson, Mississauga, ON, Canada) after gating all events on chl *a* fluorescence, *F*_*red*_, and side scatter to eliminate the signal from particles other than phytoplankton (e.g., precipitates or bacteria). The cytometer was configured with 488‐nm laser excitation and detected green and red fluorescence at 533 nm (30 nm bandwidth at half maximum emission) and >670 nm, respectively. To prevent saturation of the detectors, the fluorescence from all cultures except the cyanobacterium and the haptophytes was attenuated by 99%, using 2‐OD neutral density filters supplied by the manufacturer. The flow cytometer was calibrated daily using Spherotech 8‐peak validation beads (Becton Dickinson). Sample volumes were set to measure fluorescence intensities on 10^3^–10^5^ cells in each untreated culture. Cell concentrations were calculated from the number of cells and the volume of sample analyzed. Some cells, in most cases <2% of the population, registered no green fluorescence despite exceeding the threshold for red fluorescence, presumably because *F*_*green*_ was below instrument sensitivity. These were scored as *F*_*green*_ = 0; they were included in all nonparametric analyses including the final assessments of errors, but excluded from parametric analyses on log‐transformed data.

### Growth assays

The concentration of viable cells in selected cultures was measured using the Most Probable Number growth assay (McCrady 1915, Cochran 1950, Cullen and MacIntyre 2016). The cultures were diluted in three tiers separated by an order of magnitude (e.g., 10^−1^, 10^−2^, 10^−3^), dispensed in 5‐mL volumes into sterile Pyrex tubes (5 per dilution), and monitored for growth of the sub‐samples by measuring chl *a* fluorescence with the Turner 10AU fluorometer. Tubes were scored as positive or negative for growth and the scores used to estimate the number of viable cells (± the 95% confidence intervals) in the parent sample using look‐up tables (Blodgett 2010). These were converted to concentrations by correcting for the dilution range and the volume of the samples. The dilution ranges, criteria for scoring growth versus no growth in individual tubes, and the duration of the assays were based on protocols designed to maximize the accuracy of scoring (MacIntyre et al., unpublished data).

### Cell size

Cell size was determined for eukaryotic species from micrographs of bright‐field images collected with a Zeiss AX10 microscope and recorded with an AxioCam ERc5s camera (Zeiss, Jena, Germany). A minimum of 50 micrographs per species were analyzed in ZEN software from Zeiss, and cell size determined from cross‐sectional area as the average cell diameter (ACD), calculated as for a circle. The scaling on the micrographs was calibrated using the lines on a Reichert Bright‐Line hemacytometer (Hausser Scientific, Horsham, PA, USA).

## Results

### Effect of heat treatment and stains on cell concentrations

The effect of experimental treatment on cell concentrations of the cultures was assessed by normalizing the measured cell concentrations in all eight treatments (untreated and heat‐treated vs. unstained, and stained with FDA, CMFDA, and FDA+CMFDA) to the concentration in the untreated, unstained culture and then testing for the effect of heat treatment versus staining in two‐way ANOVA. The 3 or 5 independent tests for each species (see below) provided the replication; normalization prevented differences in untreated cell concentrations between experiments from introducing error. The two‐way analyses of effects on cell concentrations were significant (*P* < 0.05) in 18 of the 24 taxa tested. The overwhelming majority showed a significant effect of heat on cell concentrations, with six showing an effect of the stains, and 4 showing a significant interaction between heat and staining (Table 2).

**Table 2:** The effect of heat treatment and staining on the normalized concentration of cells in 24 species of phytoplankton determined by two‐way ANOVA with Heat (untreated and assumed live, vs. heat‐treated cells) and Stains (unstained vs. stained with FDA, CMFDA, and FDA+CMFDA) as factors; *n* = 3 for consistently staining taxa, and *n* = 5 for inconsistently staining taxa (see Results)

Division	Species	Significance level			Change in cell concentration (%)			
		Heat	Stains	Interaction	Heat	FDA	CMFDA	FDA+CMFDA
Cyanophyta	*Synechococcus elongatus*	–	–	–				
Chlorophyta	*Chlamydomonas* cf. sp.	**	–	–	42 ± 16			
	*Dunaliella tertiolecta*	**	–	–	24 ± 19			
	*Micractinium* sp.	–	–	–				
	*Nephroselmis pyriformis*	***	–	–	−9 ± 2			
	*Pyramimonas parkeae*	***	**	–	−12 ± 2			
	*Scenedesmus obliquus*	–	–	–				
Euglenophyta	*Eutreptiella* cf. *gymnastica*	***	**	**	−70 ± 3	−48 ± 5		−27 ± 9
Cryptophyta	*Rhodomonas salina*	***	–	–	−24 ± 4			
	*Storeatula* sp.	–	–	–				
Haptophyta	*Chrysochromulina kappa*	***	–	–	−35 ± 10			
	*Isochrysis galbana*	–	*	**				
	*Phaeocystis globosa*	*	–	–	−10 ± 9			
	*Prymnesium parvum*	–	–	–				
Heterokontophyta	*Amphiprora* sp.	*	–	–	31 ± 6			
	*Chaetoceros simplex*	–	–	–				
	*Heterosigma akashiwo*	***	*	–	−87 ± 2			
	*Thalassiosira pseudonana*	**	–	–	−4 ± 3			
	*Thalassiosira weissflogii*	*	***	*	12 ± 1			
Dinophyta	*Alexandrium andersoni*	***	–	–	−80 ± 5			
	*Amphidinium carterae*	*	–	–	−2 ± 9			
	*Heterocapsa* sp.	***	–	–	−24 ± 17			
	*Prorocentrum triestinum*	**	***	**	−51 ± 15	−59 ± 3	−54 ± 5	−55 ± 9
	*Scrippsiella trochoidea*	***	–	–	−72 ± 7			

Where tested as significant at *P* ≤ 0.05 with Tukey's a posteriori honestly significant difference procedure, the percent changes in cell concentrations versus unstained, live cells are shown as the mean ± SE. Significance levels: –, *P* ≥ 0.05; *, *P* < 0.05; **, *P* < 0.01; ***, *P* < 0.001.

The effects of heating and staining were assessed independently by a posteriori comparisons of the unstained, untreated culture with (i) heat‐treated and (ii) stained, untreated cultures using Tukey's honestly significant difference test for taxa in which the two‐way ANOVA was significant (*P* ≤ 0.05). Of the 17 species that showed an effect of heat, two chlorophytes and two diatoms showed increases in cell concentrations with heating (Table 2), presumably through disaggregation of clumps. The highest mean increases, 31% and 42%, were in *Amphiprora* and *Chlamydomonas* cf., both of which were noticeably prone to sticking to the culture glassware. There were significant cell losses in 13 species, with the highest mean losses (70%–87%) among diverse flagellates: the euglenoid *Eutreptiella*, the raphidophyte *Heterosigma*, and the dinoflagellates *Alexandrium* and *Scrippsiella*. In comparing untreated and heat‐treated samples below, we assume that any cell loss due to the heat‐treatment was nonselective with respect to staining intensity, i.e., that the frequency distributions in the surviving heat‐treated cells were representative of the population as a whole.

Of the 6 taxa in which there was a significant (*P* < 0.05) effect of stains on cell concentration, there were significant differences between the unstained, untreated cultures and the stained, untreated cultures in two: the euglenoid *Eutreptiella* and the dinoflagellate *Prorocentrum*. Both showed large mean reductions with FDA (48% and 59%, respectively) and the combined stain (27% and 55%). With a 54% mean loss after staining, only *Prorocentrum* showed significant reductions in cell concentration with CMFDA.

### Repeatability of results

Frequency distributions of per‐cell green fluorescence, *F*_*green*_, were used to test replication of staining in the three independent experiments conducted for each species, separated by 1–344 d. Data were log_10_ transformed to adjust for the negatively skewed distributions of *F*_*green*_ and reduced to an 8‐point signature consisting of the normalized mean values of log(*F*_*green*_) for each of the 2 × 4 treatments of untreated and heat‐treated versus unstained, FDA‐stained, CMFDA‐stained, and FDA+CMFDA‐stained samples. Each distribution's mean was normalized to the overall mean for the eight treatments to remove the potential effect of size (i.e., differences in fluorescence intensity per cell due differences in biovolume). For 20 of the 24 taxa tested, the mean Bray‐Curtis similarity coefficients between staining signatures for the three replicate experiments (calculated with Primer‐E software) were ≥95%. We refer to these as the consistently staining taxa. In the remaining 4 taxa, the dinoflagellates *Alexandrium* and *Amphidinium*, the haptophyte *Phaeocystis* and the chlorophyte *Pyramimonas*, the mean similarity between the three replicates was <95%. Two more experiments were conducted and analyzed for these species but in each case, there was no clear outlier. Each experimental culture satisfied stringent criteria for balanced growth prior to the measurements, so the reason for the intraspecific variability in staining pattern is unknown. We refer to these as the inconsistently staining taxa.

### Patterns of staining

**Figure 3:**
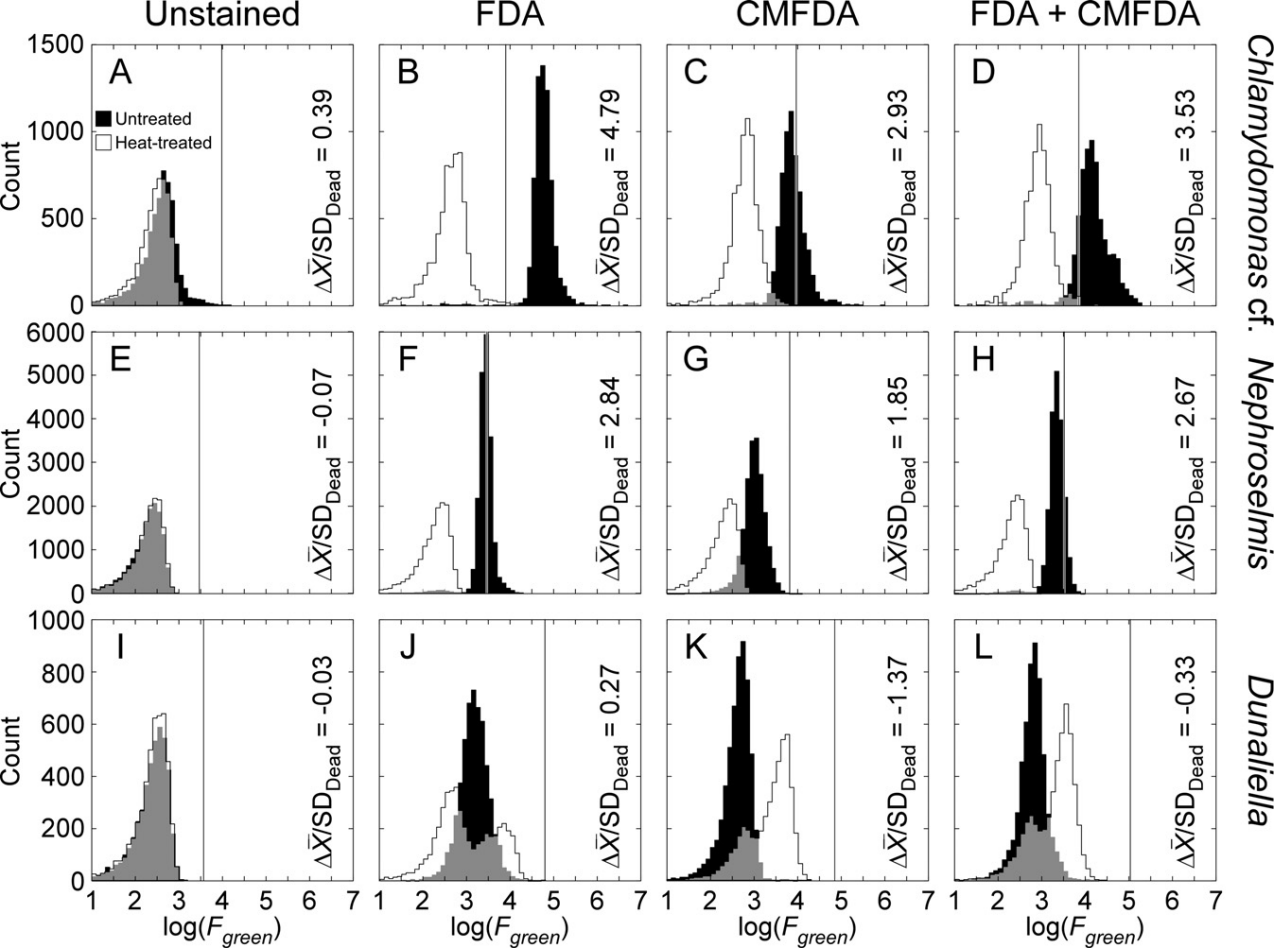
Frequency distributions of log‐transformed per‐cell green fluorescence (*F*_*green*_) in three chlorophytes: (A–D) *Chlamydomonas* cf. sp.; (E–H) *Nephroselmis pyriformis*, and (I–L) *Dunaliella tertiolecta* (Table 1). Untreated and heat‐treated cultures were assayed without stains (first column), and stained with FDA, CMFDA, and FDA+CMFDA in the following columns. Gray areas indicate overlap between distributions of untreated and heat‐treated cultures. The vertical lines in each panel are the $F_{Threshold}^{Dead}$ values established from the mean + 3SD of log(*F*_*green*_) of the heat‐treated populations. The separation between distributions, as $\Delta \overset{¯}{X}/{\text{SD}}_{Dead}=\left[\bar{\text{log}(F_{Live})}-\bar{\text{log}(F_{Dead})}\right]/{\text{SD}}_{Dead}$, is shown on each panel.

The results for one replicate of each of three consistently staining chlorophytes are shown in Figure 3. (The results for one replicate of each species tested are shown in Figs. S1–S6 in the Supporting Information). There was minimal, if any, difference between the frequency distributions of green autofluorescence in unstained, untreated versus heat‐treated cells (median $\Delta \overset{¯}{X}/{\text{SD}}_{Dead}$ = −0.03; Fig. 3, A, E, I). *Chlamydomonas* cf. showed a pattern consistent with expectations for a vital stain (Fig. 3, B–D): the signal from heat‐treated, stained cells was similar to that for unstained cells and staining intensity for untreated cells was higher than for heat‐treated cells. Separation was higher for FDA ($\Delta \overset{¯}{X}/{\text{SD}}_{Dead}$ = 4.79) than for CMFDA and FDA+CMFDA ($\Delta \overset{¯}{X}/{\text{SD}}_{Dead}$ = 2.93 and 3.53, respectively). Using the thresholds calculated from heat‐treated cells (eq. (2)), the rates of false negative classification for untreated cells were 1% for FDA, 64% for CMFDA, and 33% for FDA+CMFDA. *Nephroselmis* followed similar trends but the separation between untreated and heat‐treated cells (Fig. 3, F–H) was less (1.85 ≤ $\Delta \overset{¯}{X}/{\text{SD}}_{Dead}$ ≤ 2.84) and the rates of false negatives were commensurately higher: 65% for FDA, 97% for CMFDA, and 79% for FDA+CMFDA. The staining pattern for *Dunaliella* was completely inconsistent with expectation for vital stains. When stained, the distributions for heat‐treated cells were bimodal and the upper end of the distributions exceeded those of the untreated cells (Fig. 3, J–L). Calculated values of $\Delta \overset{¯}{X}/{\text{SD}}_{Dead}$ ranged from −1.37 to 0.27. The threshold classification cannot be applied rigorously to these data as per‐cell fluorescence of the heat‐treated cells was not normally distributed. However, as the upper end of the distributions of heat‐treated cells were consistently higher than those of untreated cells, the rate of false negatives for *Dunaliella* can unambiguously be assessed as 100% for all three stains.

**Figure 4:**
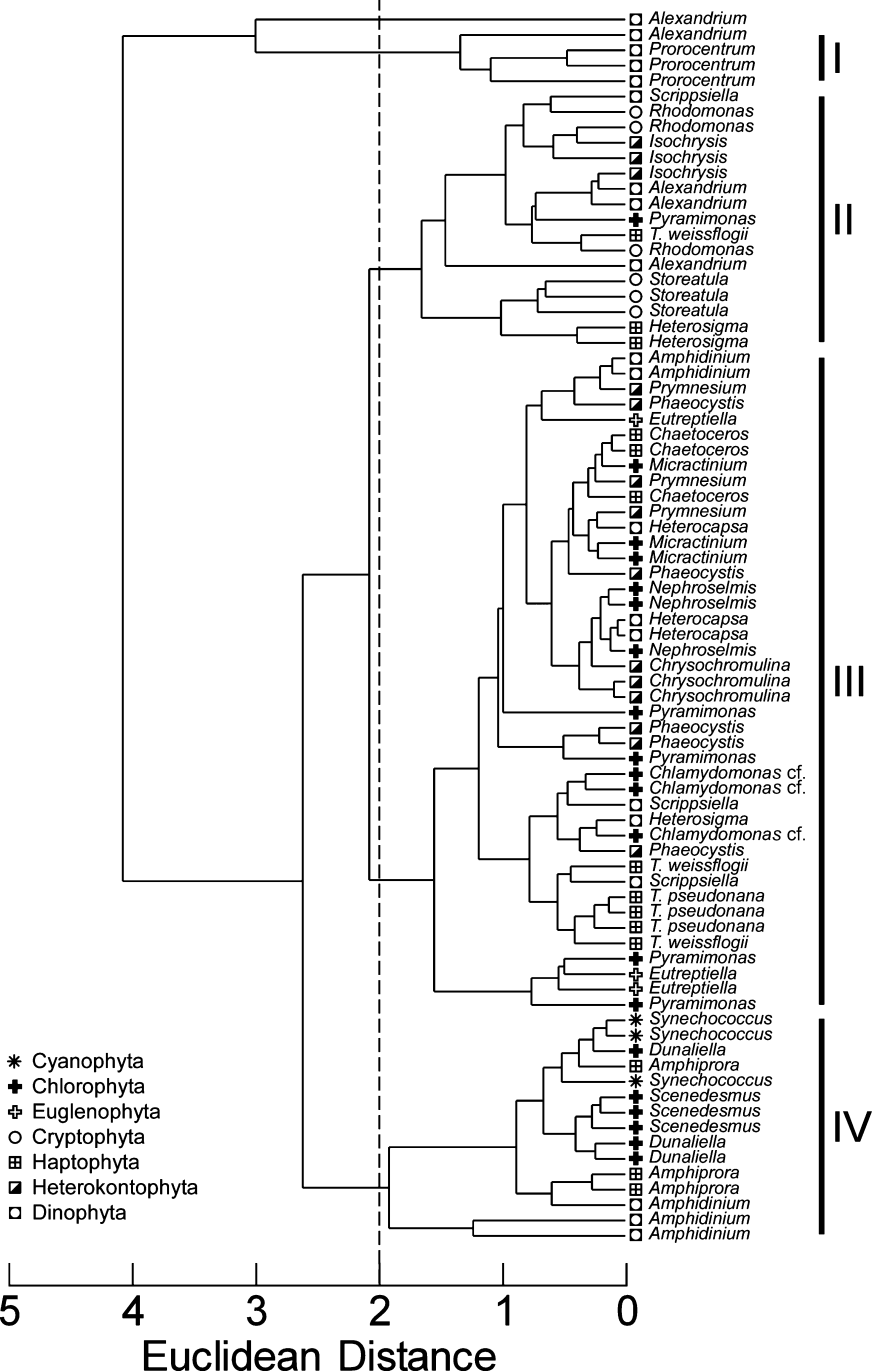
Dendrogram showing the results of an hierarchical cluster analysis of the parameter $\Delta \overset{¯}{X}/{\text{SD}}_{Dead}$ for each of three staining treatments (FDA, CMFDA, and FDA+CMFDA) for 24 species of phytoplankton. There are 3 or 5 replicates per species, depending on the similarity of their mean staining signatures (see text). At a Euclidean distance of 2 (dashed line) there are four clusters (I–IV) and one replicate of *Alexandrium* that did not cluster in any of them.

To facilitate the classification of common responses, the patterns in staining among the 24 species tested were analyzed by reducing the distributions for each replicate to a 3‐point signature derived from the values of $\Delta \overset{¯}{X}/{\text{SD}}_{Dead}$ calculated for live versus heat‐treated cells for each of the three stain treatments. For each replicate of each sample, the values of $\Delta \overset{¯}{X}/{\text{SD}}_{Dead}$ for FDA, CMFDA, and FDA+CMFDA were normalized to the mean of the three values. The signatures of each sample were then compared by hierarchical cluster analysis using Primer‐E software. All samples except one replicate of *Alexandrium*—one of the inconsistently staining species—were grouped in four clusters at an Euclidean distance of 2 (Fig. 4). Replicates of all of the consistently staining taxa were classified in the same cluster, with the exception of *Heterosigma* and *T. weissflogii*, which were divided between Clusters II and III. Recall that the classification of the consistently versus inconsistently staining taxa was based on the mean log(*F*_*green*_) of all 8 eight treatments, not $\Delta \overset{¯}{X}/{\text{SD}}_{Dead}$. Both *Chlamydomonas* cf. and *Nephroselmis* (Fig. 3) were classified in Cluster III; *Dunaliella* (Fig. 2) was in Cluster IV. Of the inconsistently staining species, *Alexandrium* was classified as one outlier and in Clusters I and II, *Amphidinium* was classified in Clusters III and IV, *Pyramimonas* was classified in Clusters II and III, and *Phaeocystis* was classified solely in Cluster III. When summarizing results for clusters of species, the 6 species comprising the 4 inconsistently staining taxa and the 2 taxa that were grouped in more than one cluster are referred to as “Mix.”

**Figure 5:**
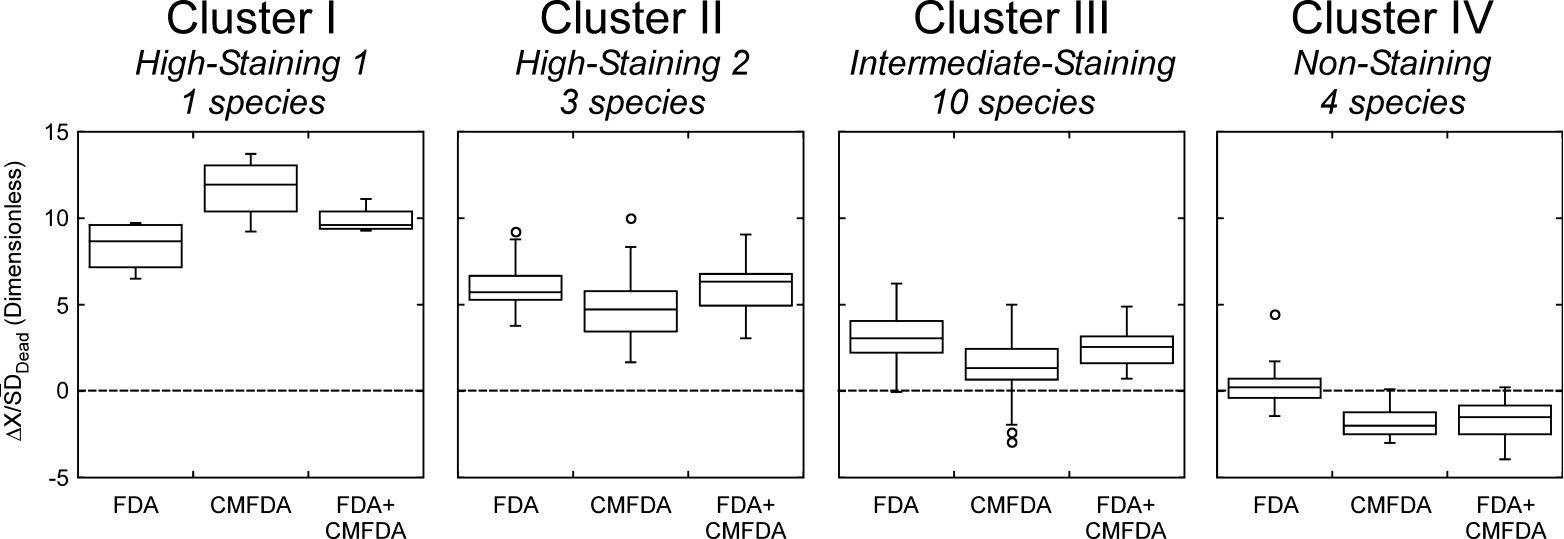
Box plot of the parameter $\Delta \overset{¯}{X}/{\text{SD}}_{Dead}$ for results using each of three staining treatments (FDA, CMFDA, and FDA+CMFDA), grouped according to the cluster analysis in Figure 4. Distributions are for 3 or 5 replicates for consistently staining and inconsistently staining taxa, respectively. The number of species refers to the consistently staining species in each cluster. The parameter describes the difference between staining intensities in untreated and heat‐treated populations. The dashed line at $\Delta \overset{¯}{X}/{\text{SD}}_{Dead}$ = 0 indicates complete overlap of distributions: the higher the value, the more distinct the staining signatures are. Note that some values in Cluster IV are negative: the mean of log‐transformed fluorescence in the dead populations was higher than in the live populations for these samples.

Variations in the separation parameter $\Delta \overset{¯}{X}/{\text{SD}}_{Dead}$ by cluster are illustrated in Figure 5, suggesting a general characterization for each of the four groups. The taxa in Clusters I and II had high values for all three stains and should have minimal numbers of false negatives. We refer to these as High‐staining 1 and 2. The ranges of separation were lower in Cluster III (Intermediate‐staining), ranging from values high enough to ensure minimal numbers of false negatives to values of zero (i.e., no discriminatory power; Fig. 2). There was very little separation for the four species in Cluster IV (referred to as nonstaining) and the values were negative for CMFDA and FDA+CMFDA. The stains would have little if any discriminatory power in this cluster (e.g., *Dunaliella*, Fig. 3). The distribution of species between the low‐ and high‐staining clusters was ataxonomic: of the divisions represented by more than 2 species, all were distributed between different clusters (i.e., had different patterns of staining). The dinoflagellates were distributed in Clusters I–IV and the chlorophytes, haptophytes, and heterokontophytes were each classified in Clusters II–IV.

### Quantification of errors

**Figure 6:**
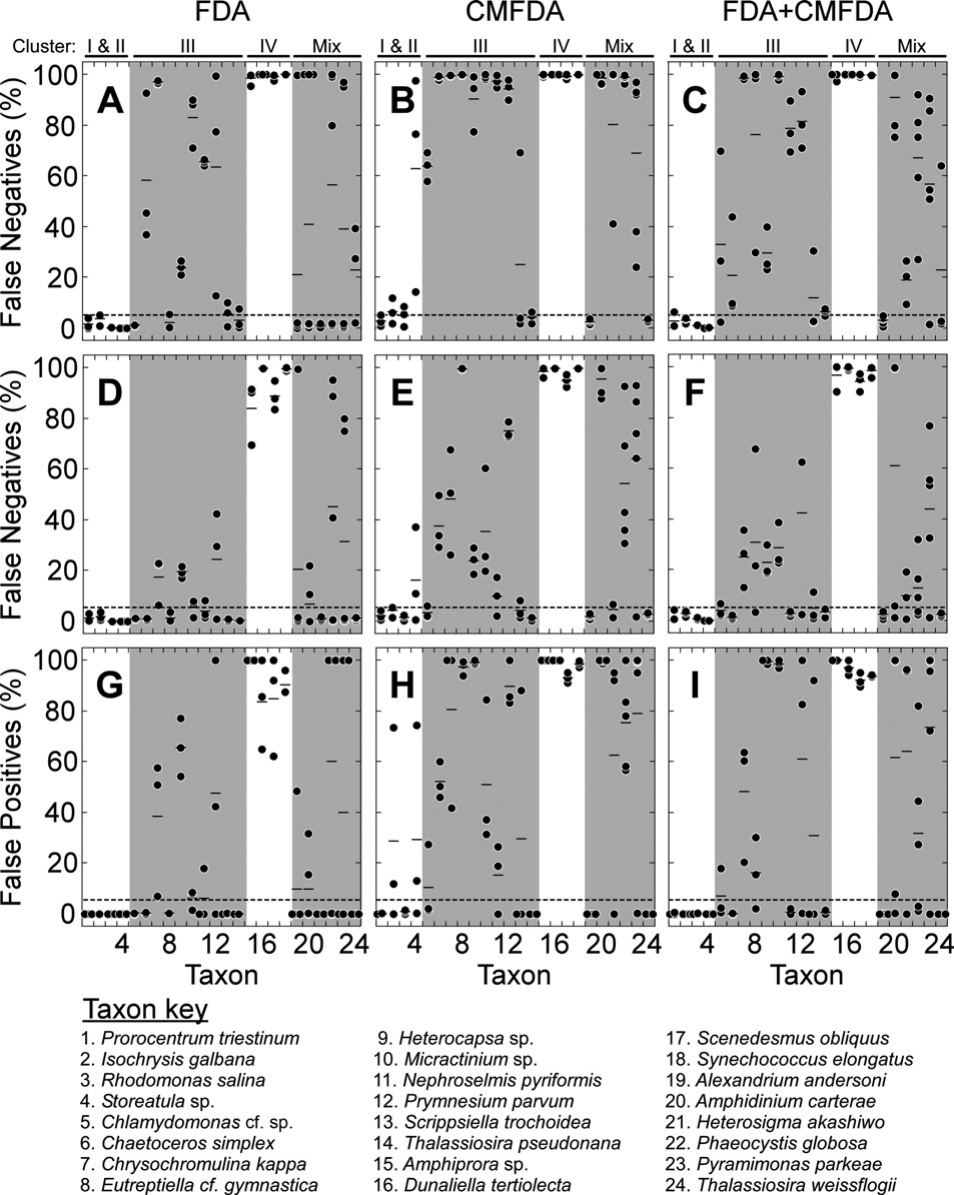
Dot plots of the rates of false negative and false positive results with FDA, CMFDA and FDA+CMFDA in the columns, as determined with three different statistical protocols, in rows. (A–C) False negative errors based on the parametric statistically‐not‐dead threshold defined as $\text{log}\left(F_{Threshold}^{Dead}\right)=\bar{\text{log}(F_{Dead})}+3{\text{SD}}_{Dead}$ (eq. (2)). (D–F) False negative error rates using a comparable nonparametric $F_{Threshold}^{Dead}$ set to the 95th percentile of *F*_*Dead*_. This fixes the rate of false positive errors at 5%. (G, H) False positive errors using the statistically‐not‐alive threshold $F_{Threshold}^{Live}$ = 5th percentile of *F*_*Live*_. This fixes the rate of false negative errors at 5%. Taxa are grouped by clusters from Figure 4; “Mix” consists of the inconsistently staining taxa and taxa that were grouped in more than one cluster. Horizontal bars are the means for 3–5 replicates for each species and dashed lines indicate the 5% error level.

The rates of false positive and false negative errors were calculated using the parametric statistically‐not‐dead threshold, $F_{Threshold}^{Dead}$ (eq. (2)), and are illustrated by cluster in the top row of Figure 6. The rates of false positives (data not shown) were uniformly low, <2%, but higher than the value of 0.14% predicted for a normal distribution (Sokal and Rohlf 1981). As inferred from the distributions of $\Delta \overset{¯}{X}/{\text{SD}}_{Dead}$ (Figs. 5), false negative errors were uncommon in the four taxa in the High‐staining Clusters I and II, and for *Alexandrium*, which was a mix of I and II. Rates of false negatives were higher, with many approaching 100%, in Intermediate‐staining Cluster III and all were close to 100% in Non‐staining Cluster IV. Of the 24 taxa tested, 6, 3, and 4 were classified accurately (≤5% false negatives, with the mean for all replicates corrected for cell loss where significant—see Tables 2 and 3) for FDA, CMFDA, and the combined stain, respectively (Table 3).

**Table 3:** Proportions of taxa that were accurately classified as live versus dead using different classification thresholds for the three stain treatments. Untreated cells are assumed to be live and heat‐treated cells are assumed to be dead (see text). False negatives are live cells that are classified as dead and false positives are dead cells that are classified as live. Each classification threshold imposes an error rate on either false positives or false negatives; the accuracy criterion scores the tests for each species according to the type of error that is not constrained. The accuracy is assessed first from the mean proportion of false negatives and positives for the 3–5 replicates, and then from the subset of those that did not have significant cell loss on staining (*P* > 0.05; Table 2)

Classification threshold	Imposed error rate	Accuracy criterion	Accurate classification/24 taxa		
			FDA	CMFDA	FDA+CMFDA
Statistically‐not‐dead					
Parametric $\text{log}\left(F_{Threshold}^{Dead}\right)=\bar{\text{log}(F_{Dead})}+3{\text{SD}}_{Dead}$	False positives = 0.14%^a^	False negatives ≤5%^a^	8	4	5
		Of those, taxa with no cell loss on staining	6	3	4
Nonparametric $F_{Threshold}^{Dead}$ = 95th percentile of *F*_*Dead*_	False positives = 5%	False negatives ≤5%	12	9	11
		Of those, taxa with no cell loss on staining	10	8	10
Statistically‐not‐Alive					
Nonparametric $F_{Threshold}^{Dead}$ = 5th percentile of *F*_*Live*_	False negatives = 5%	False positives ≤5%	11	5	9
		Of those, taxa with no cell loss on staining	9	4	8

a The expected rate of false positives is 0.14%. Observed values were all <2%. Data are reported as the number of taxa meeting each criterion. A total of 24 taxa were tested.

The classification in Figure 6, A–C is based on statistical descriptions of staining in the heat‐treated populations, with an assumption of univariate normality that is not met in some cases (see Figs. 3, J–L and  S1–S6); the exclusion of cells registering no green fluorescence (see Materials and Methods) introduced additional uncertainty. These issues were obviated by applying the alternative, nonparametric, statistically‐not‐dead classification based on an $F_{Threshold}^{Dead}$ set to the 95th percentile of the heat‐treated cells' distribution. The rate of false positives was thereby fixed at 5%, and the cells that registered no green fluorescence were appropriately included in the distributions. Rates of false negatives with this classification are shown in the middle row of Figure 6 (see also Figs. S1–S6). They are lower than those reported for the parametric test (Table 3) only because the classificatory threshold is less stringent, allowing 5% false positives versus an expected level of 0.14% for the parametric approach (see Fig. 2C). As with the results of the parametric approach (Fig. 6, A–C), the rates of false negative misclassification with the nonparametric approach (Fig. 6, D–F) were very low for the taxa in the two High‐staining groups, Clusters I and II, but variable and frequently high for those in Intermediate‐staining Cluster III and for inconsistently staining taxa in the “Mix” group. Rates of false negatives were uniformly high for taxa in Non‐staining Cluster IV. Even at this relaxed level of stringency, accepting 5% false positives versus <2% in the parametric classification, fewer than half of the taxa tested met the criterion of ≤5% false negatives (Table 3). As before, FDA outperformed CMFDA and was equivalent to the combined stain (10, 8, and 10 accurate classifications, respectively, out of 24 taxa tested).

Use of the 95th percentile of the heat‐treated cells' distribution to define $F_{Threshold}^{Dead}$ in a statistically‐not‐dead approach sets the false positives at a low level, forcing false negatives to increase when there is overlap between the distributions. In contrast, use of a statistically‐not‐alive approach allows the rate of false negatives to be set at a nominal low value by defining $F_{Threshold}^{Live}$ from the live cells' distribution. When the 5th percentile of fluorescence intensity is used, the rate of false negatives is by definition 5%. The effect of the alternate criterion on false positives is shown in the bottom row of Figure 6. The rate of false positives was well above 5% for many of the taxa in Intermediate‐staining Cluster III and Non‐staining Cluster IV but also in many of the “Mix” group in which $\Delta \overset{¯}{X}/{\text{SD}}_{Dead}$ was low (see Fig. 2C). The taxa with elevated rates of false negatives when classified with $F_{Threshold}^{Dead}$ (Fig. 6, A–F) also had high rates of false positives when classified with $F_{Threshold}^{Live}$ (Fig. 6, G–I), a reflection of the fact that their frequency distributions overlapped, so no threshold can eliminate error. Consistent with the expectation of some sort of reciprocity between false positive and false negative errors as thresholds change, the proportion of taxa that met the criterion of ≤5% false positives with the nonparametric statistically‐not‐alive approach was comparable to the proportion of false negatives with the corresponding statistically‐not‐dead criterion (Table 3). Of the 24 taxa tested, 9, 4, and 8 were accurately classified for FDA, CMFDA, and the combined stain, respectively (Table 3). The important difference with the use of $F_{Threshold}^{Live}$ is that the rate of false negatives is fixed at 5%, which confers confidence in environmental protection, albeit at the expense of requiring the ballast water management system to perform better than required by regulations in order to pass the discharge standard.

### Validation of heat‐killing

To determine if heat‐treated cells were indeed dead, as is assumed for our analyses, we tested the five species in Nonstaining Cluster IV—those for which results were most aberrant, conceivably due to ineffective heat‐treatment. Effectiveness was assessed by estimating the concentrations of viable cells in live and heat‐treated samples using Most Probable Number growth assays incorporating a grow‐out period of >20 d. Viability was assumed to reflect vitality under the experimental conditions (see Discussion).

**Figure 7:**
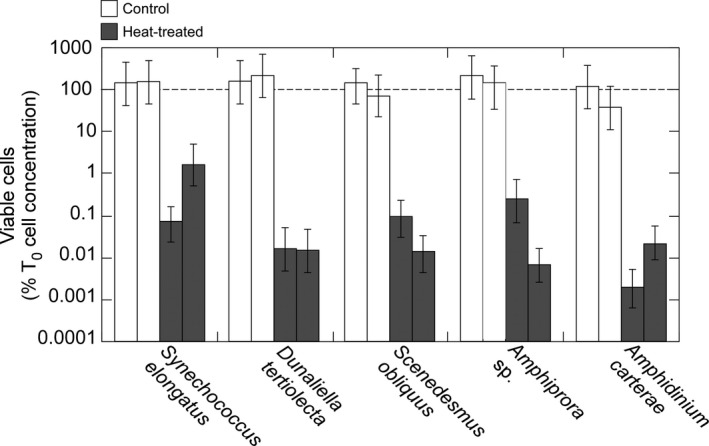
Relative concentrations of viable cells in control (untreated, live) and heat‐treated cultures of the nonstaining taxa in Cluster IV (Fig. 4). Note log scale. Viability was estimated by the Most Probable Number assay and normalized to initial cell concentration, measured with a flow cytometer. Error bars are 95% confidence intervals of the estimate. Each taxon/treatment was analyzed in duplicate. The dashed line indicates equivalence between the estimate of viable cells and the measured cell concentrations.

For each of the five taxa, the 95% confidence intervals of the estimated concentrations of viable cells included the initial cell concentration, measured with a flow cytometer (Fig. 7). Similar results were found with another 10 of the species (data not shown); the remaining 9 species were not tested. The untreated cells were therefore living. In contrast, heat treatment reduced the concentrations of viable cells to 0.002%–1.6% (mean, 0.04%) of the initial cell concentration (Fig. 7). The heat‐treated cells were therefore overwhelmingly non‐viable and, by inference (see Discussion), dead. Thus, we reject the hypothesis that the failure of the stains to discriminate between the untreated and heat‐treated cells in Nonstaining Cluster IV was because the two populations were not uniformly vital and uniformly nonvital respectively. More broadly, we assume that heat‐treated phytoplankton in all of our experiments were dead.

### Interspecific variability in classification thresholds

**Figure 8:**
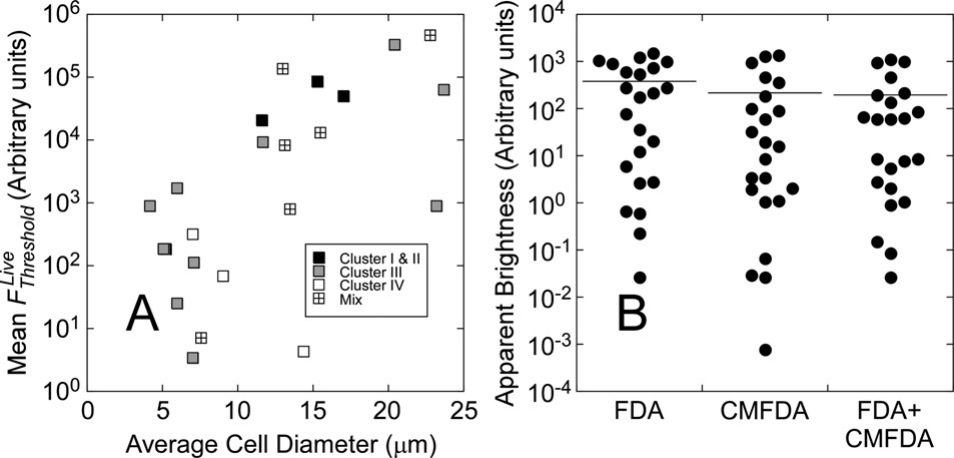
(A) The classification threshold for FDA+CMFDA based on the staining of live cells, $F_{Threshold}^{Live}$ (5th percentile of stain intensity in live cells), as a function of average cell diameter in 23 species of eukaryotic phytoplankton (Table 1). Data have been reconciled between species to account for the use of 2‐OD filters with all cultures except the haptophytes. Note log scale. Cells with fluorescence higher than the threshold are classified as living. Data are presented by classificatory cluster (Fig. 4): “Mix” refers to taxa that were distributed across more than one cluster. Note that the minimum fluorescence indicative of a living cell varies by several orders of magnitude, but not solely as a function of cell size: thresholds for cells of similar diameter can vary by more than two orders of magnitude. (B) Dot plots showing apparent brightness predicted for observation with epifluorescence microscopy, estimated as $F_{Threshold}^{live}$ normalized to cross‐sectional area for cells stained with FDA, CMFDA, and the combined stain. Note log scale. The horizontal lines are the arithmetic means. Interspecific variability in apparent brightness at the classificatory threshold approaches 5 orders of magnitude between species for FDA and the combined stain and is greater than 6 orders of magnitude for CMFDA.

A single threshold of *F*_*green*_ for discriminating live from dead cells cannot be applied to a community of phytoplankton: for the 23 eukaryotic species tested here, the mean value of the nonparametric $F_{Threshold}^{Live}$ varied by five orders of magnitude (Fig. 8A), showing a correlation with cell size (*R* = 0.58, *n* = 23, *P* < 0.001), but with wide variation around the trend. For instance, *Prymnesium*,* Micractinium*, and *Scenedesmus* are all 7 ± 1 μm in ACD (Table 1) but have mean values of $F_{Threshold}^{Live}$ that vary by >93*x*. Differences between cells of different sizes were assessed by normalizing $F_{Threshold}^{Live}$ to cross‐sectional area. This is an estimate of apparent brightness, for example as observed using epifluorescence microscopy. The apparent brightness of cells at the classification threshold varied by almost 5 orders of magnitude for FDA and the combined stain and by more than 6 orders of magnitude for CMFDA (Fig. 8B).

## Discussion

The metabolic pathways leading to cell death in the phytoplankton are not clear and there is no single criterion that can be used to discriminate dead from living cells (reviewed by Franklin et al. 2006, Berges and Choi 2014). A fairly broad diversity of approaches for classifying vitality or mortality has been described, but in a variety of comparisons, none has been judged to out‐perform the vital stain FDA (Reynolds et al. 1978, Veldhuis et al. 1997, Reavie et al. 2010, Peperzak and Brussaard 2011, Zetsche and Meysman 2012, Imase et al. 2013), despite its well‐recognized limitations. The variability of staining intensity between taxa (Dorsey et al. 1989, Agustí and Sanchez 2002, Garvey et al. 2007, Reavie et al. 2010, Peperzak and Brussaard 2011) and with growth conditions in a single taxon (Selvin et al. 1988, Gilbert et al. 1992, Brookes et al. 2000, Garvey et al. 2007) are well‐documented for FDA. To compensate for these shortcomings, Steinberg et al. (2011) investigated the use of other vital stains with fluorescence emission spectra similar to FDA. Supported by the results of preliminary trials on natural assemblages of protists and cultures of microalgae, they proposed the simultaneous and combined use of FDA with CMFDA, a thiol probe‐reactive probe reported to have better cellular retention (Steinberg et al. 2011, citing Poot et al. 1991).

To date, the most comprehensive analysis of differences in staining between living and dead phytoplankton has been by Peperzak and Brussaard (2011). The study reported live/dead staining ratios for FDA and CMFDA but did not explicitly quantify error rates, nor did it test the combined stain. Although what they characterized as “preliminary trials” on FDA, CMFDA, and the combination of the two supported the conclusion of Steinberg et al. (2011) that “combining fluorescent stains is a robust, powerful tool that can be optimized for the species present at each location,” FDA+CMFDA had yet to be validated with systematic and comprehensive testing using demonstrably viable and nonviable phytoplankton, states assumed to reflect vitality and nonvitality in the context of the experiments, nor with strict taxonomic separation, conditions that cannot be met with natural assemblages.

### Quantifying the efficacy of vital stains

This study focused on a rigorous assessment of vital stains using quantitative measures of staining intensity, objective thresholds for discrimination, and test populations that have been validated as being viable or nonviable, and by inference, classifiable as live or dead (Fig. 7). Our assumption that loss of viability from heating to 50°C, the killing method used by Steinberg et al. (2011), reflects loss of vitality at the time of treatment is based on Occam's Razor. Although we cannot rule out that heat‐treated cells were vital but nonviable, the often large loss of detectable cells immediately after heat treatment (Table 2) and microscopic observation of cells with condensed cytoplasm and/or distorted morphologies after heat treatment suggest that the cells rendered nonviable by heating were killed outright.

Use of flow cytometric measurements of per‐cell fluorescence intensity allowed direct enumeration of false positive and false negative errors in each experiment. Applying a criterion for accuracy that is not overly harsh—an average across replicates of ≤5% false negatives plus ≤5% false positives, based on nonparametric criteria that are not subject to the uncertainties of parametric analysis—we found that the best performing of the three assays, FDA, is an effective indicator of vitality in only 12 of 24 species, with a score of 11/24 for the second best assay, FDA+CMFDA (Table 3). However, in the context of testing for the concentrations of living cells against a regulatory threshold, these counts are further reduced because two of the species with good separation of living and heat‐treated (dead) cells, *Eutreptiella* and *Prorocentrum*, had significant losses of cells when stained. The mean losses of live cells were 48% and 59%, respectively, after treatment with FDA, and 27% and 55% after treatment with the combined stain (Table 2). Staining tests for either species would therefore significantly underestimate the concentrations of living cells, with a corresponding increase in false negative errors that are accounted for in Table 3. Consequently, we conclude that the use of vital stains gave acceptably accurate results (i.e., ≤5% false negatives plus ≤5% false positives, averaged across replicates, and no significant loss of cells on staining) for at best only 10 species out of 24, (i.e., 42% of the taxa tested). Combination of CMFDA with FDA did not improve the performance of FDA alone. Adams et al. (2014) also concluded that addition of CMFDA to FDA did not alter the classification of live versus dead cells from a broad range of taxonomic groups in natural samples.

Of the taxa that did not meet the criteria of both ≤5% false positives and ≤5% false negatives, results were consistent with expectations based on separation of staining intensity between untreated and heat‐treated cells (Fig. 5). In Cluster III, there was significant overlap between the frequency distributions of untreated and heat‐treated cells, as exemplified by the response of *Nephroselmis* to CMFDA in Figure 3; error rates were high and variable (Fig. 6). In Non‐staining Cluster IV, the vital stains method failed because there was near‐total overlap or higher staining in heat‐treated cells than in untreated ones, as exemplified by the response of *Dunaliella* to all stains in Figure 3 (see also Figs. S1, S2, S4, and S6). This is inconsistent with the assumptions of vital stains. We verified that the overlap in frequency distributions in four nonstaining taxa was not an artifact, mistakenly comparing cultures that were both alive or both dead, by comparing cell counts in live and heat‐treated cultures with estimates of viable cells from a growth‐based assay. The results confirmed that the untreated cells were indeed living and that the proportion of cells in the heat‐treated cultures that remained capable of growth was 10^−2^ to 10^−5^ of the initial population. The failure to stain is therefore intrinsic to these cultures under the conditions of balanced growth used in the experiments. The fluorescence distributions of heat‐treated cells from Cluster IV were typically bimodal, with an upper peak that had higher fluorescence than the untreated controls (e.g., *Dunaliella* in Fig. 3). Heat‐activation of the esterases/thiols and increased catalytic rates or decreased permeability of the cell membrane to the fluorescent products seems improbable at 50°C. A more likely alternative is that heat treatment increased permeability of a cell membrane that was otherwise highly impermeable to the acetylated stains, facilitating entry of the substrates into a fraction of the cells without causing sufficient disruption to prevent accumulation of the fluorescent product. If correct, this suggests that a fundamental assumption of the vital stain method, that the substrate can enter cells freely (Rotman and Papermaster 1966), does not apply to a significant proportion of the species tested.

In addition to the taxa in Cluster IV that had consistently poor or no staining, the six species in the “Mix” group showed inconsistent patterns of staining and in the extreme cases showed results that varied from ≤5% to >95% rates of false negatives in repeated independent experiments with the same stain. We are unable to explain why our carefully controlled procedures yielded excellent replication in many species and poor repeatability in a few: as the results for two additional replicates indicated, inconsistent results were attributable to the species, not anomalies in the experimental routine. All the cultures were maintained in exponential, nutrient‐replete growth, which precludes nutrient stress (cf. Brookes et al. 2000, Garvey et al. 2007) as a source of the variability. There were no significant differences in generation time nor cell size between consistently and inconsistently staining taxa and all staining was conducted in a window 4–6 h after the onset of the dark period, which makes it unlikely that differences in the cell cycle in phased division might be responsible (cf. Gilbert et al. 1992).

Because the “Mix” species constitute one quarter of those tested, poor repeatability introduces a second, significant level of uncertainty in a stain‐based assessment of vitality that is different from the consistent heterogeneity of staining in the Intermediate‐staining taxa in Cluster III. Both of these responses are evident in cultures maintained in balanced growth, a condition that was essential to ensure that all cells in the cultures were homogenous and in active growth (i.e., alive) and that tests could be fully replicated in independent experiments. A third source of variability in staining, which was not therefore addressed in this study, is well‐documented in the literature: uncertainty arising from any variation in the growth conditions experienced by the culture. Given the changes in staining intensity inherent in unbalanced growth, is very likely that the discriminatory ability of the stains will also show intraspecific variability with environmental preconditioning to light history, temperature and/or the availability of nutrients.

### Implications for assays of natural assemblages

There was no interpretable pattern in the distribution of taxa between High‐, Intermediate‐, and Nonstaining groups, so our results provide no basis for targeting the vital stains method at consistent fractions of a natural phytoplankton assemblage (e.g., all taxa present except dinoflagellates). We have also shown in Figure 8 very high interspecific variability in the magnitude of the statistically‐not‐alive discriminatory threshold, parameterized either as per‐cell fluorescence intensity, the criterion used to discriminate in flow cytometry, or as apparent brightness when normalized to cross‐sectional area. The latter is a diagnostic that is relevant to epifluorescence microscopy, the method recommended for use in the validation of ballast water treatment (EPA 2010). Apparent brightness at the discrimination threshold varied by almost 5 orders of magnitude between species, suggesting that analysis of mixed populations using epifluorescence microscopy would require the assignment of appropriate signal thresholds to taxa before classifying the cells as dead or alive. This would require, in effect, that the live‐versus‐dead fluorescence responses of all species in a natural sample be known a priori by a trained operator, who would apply the appropriate visual thresholds. Systematic comparison of assay samples with heat‐killed controls could help, but it would not be practical to compile well‐resolved frequency distributions for live versus heat‐killed taxa using microscopy. And, as we have shown in this study based on cytometric measurements of cultures grown under controlled, benign conditions, even confidently assigned classification thresholds yielded significant error rates in a majority of the species. Changes in staining patterns associated with growth conditions would increase rates of error in any species‐specific classification.

While the comparison of viable versus nonviable samples, assumed to reflect living and dead cells, can be done with confidence using cultures in balanced growth, it is problematic for natural populations because there is no assurance that all cells are living (Paerl 1978, Veldhuis et al. 2001, Agustí and Sanchez 2002, Agustí 2004). For high‐ and intermediate‐staining species, the presence of dead cells in an untreated natural population will lead to underestimation of $F_{Threshold}^{Live}$ and a corresponding increase in false positives. Nonstaining species such as those in Cluster IV cannot be assessed accurately with vital stains, no matter what the approach.

### Classification thresholds and environmental protection

As illustrated in Figures 2 and 3, errors are unavoidable when staining distributions of living and dead cells overlap. However, as shown in Table 3, nonparametric classification thresholds can be set to prescribe low rates, e.g., 5%, of either false positive or false negative errors, each at the expense of the other. The statistically‐not‐dead threshold treats the staining of heat‐treated cells much as an analytical blank: it allows false‐positive errors, the classification of dead cells as live, to be set at a low rate. The statistically‐not‐alive criterion is based on the staining of living cells: it allows false negative errors (living cells classified as dead) to be set at a low rate that is suitable for environmental protection, but at the expense of increasing false positives. Importantly, if it can be assumed that the $F_{Threshold}^{Live}$ is above the limit of detection of the observation method, thereby ensuring that the rate of false negatives is appropriately constrained, the statistically‐not‐alive criterion generates maximum estimates of living cells. Then, tests indicating compliance with regulations provide evidence that the concentration of living cells is indeed below the regulatory standard. However, achieving this level of protection comes at the expense of requiring the treatment system to perform better than required by regulations in order to meet the discharge standard.

### Vital stains and the testing of ballast water treatment

Considering methods for discerning living from dead cells in the 10–50 μm size range in the testing of freshwater ballast discharge, Reavie et al. (2010) proposed four criteria to evaluate the efficacy of a test for vitality. It should be:

1. quantitatively precise with a lack of false positives or false negatives at time of analysis;
2. consistent across taxa;
3. consistent across [ballast water] treatment types; and
4. practical for rapid, high‐throughput, on‐site assessments.

We have demonstrated that FDA, CMFDA, and the combination of the two fail Criteria 1 and 2: rates of error can be high and accuracy varies greatly across taxa. Our evaluation of vital stains focused on the core assumption of the method — that it discriminates live from dead cells — rather than on consideration of a range of ballast water treatment types, including biocides (see, e.g., Gregg et al. 2009). It is possible that for some species, the staining patterns for intact cells killed by a method other than heat treatment would show more separation from those of live cells, and therefore lower rates of error. This could make it less complicated to evaluate some ballast water treatment technologies, but it would also be an explicit demonstration that the vital stains do not provide consistent results for all dead cells and thus fail Criterion 3. Regardless, we have previously shown that, if cell viability and thus invasive potential is the criterion (see First and Drake 2013, Cullen and MacIntyre 2016), the combined stain fails Criterion 3 because vital stains do not detect the loss of viability after treatment with UV‐C irradiation: a high proportion of cells that are rendered nonviable after treatment with UV‐C are scored as live, even using taxa in which stains accurately reflect heat‐killing (Wright and Welschmeyer 2015, MacIntyre et al. unpublished data). This is because the primary targets of UV‐C damage are nucleotides (Sinha and Hader 2002) rather than the esterases/thiols and membrane integrity that are assayed by these vital stains. Although the ease of use, Criterion 4, does favor use of FDA and CMFDA, we conclude, based on quantitative analysis of the responses of 24 species of phytoplankton cultured and tested under carefully controlled conditions, that the stains cannot be assumed a priori to be reliable and accurate for all species of phytoplankton.

## Conclusions

Defining accuracy as less than 10% error in the discrimination of live from heat‐killed cells (i.e., <5% false negatives plus <5% false positives), we have demonstrated that the vital stains FDA and CMFDA, used alone or in combination, could not accurately discriminate live from dead cells in the majority of 24 species of cultured phytoplankton tested in quantitative, replicated, and rigorously controlled experiments. Errors were unavoidable when the staining intensities of live and dead cells overlapped. When the discrimination threshold was based on an upper limit of the signal from dead cells (statistically‐not‐dead criterion), rates of false negative errors were high. Misclassification of living cells as dead can compromise protection of the environment in the testing of ballast water discharge. If instead the threshold were based on the lower limit of signal from live cells (statistically‐not‐alive criterion), false negative results would be kept low, but at the expense of high rates of false‐positive errors. There were 9 and 8 species with error rates <10% (Table 3) for FDA and the combined stain, respectively, similar to the numbers classified with the statistically‐not‐dead criterion. Regardless, any living, stained cells that are undetectable because the assay method, e.g., microscopy, is not sensitive enough, will record false negative results.

Independent replication of results was good for the majority of species tested, but 6 showed poor repeatability. The effectiveness of the stains in discriminating between live and dead cells was ranked as FDA ≥ FDA+CMFDA > CMFDA. Staining patterns, and thus the ability of stains to discriminate between live and dead cells, could not be related to taxonomic affinities, with members of the Chlorophyta, Haptophyta, Heterokontophyta, and Dinophyta ranging from high discriminatory power to little or none. Large differences in the discriminatory thresholds of fluorescence intensity between species complicate the application of quantitative live‐dead classification thresholds for natural communities of phytoplankton. We conclude that the stains are strictly reliable for use only with cultures or mixed assemblages for which their performance has already been verified.

We thank Shannah Rastin, Jessica Miller, Magda Waclawik, Anna Haverstock, Rhea Newman, and Jessica Hurtubise for culture maintenance and performing the testing, and Brian Petri for comments on the manuscript. We thank two anonymous reviewers and the editor, Dr. Jackie Collier, for comments that led to improvements of the manuscript. This work was supported by a Collaborative Research and Development Grant (Project 445451‐12) from the Natural Sciences and Engineering Research Council of Canada and Trojan Technologies, with supplementary funding from the Strategic Cooperative Education Incentive Program of Nova Scotia and Trojan Technologies.

## Supporting information


**Figure S1.** Frequency distributions of log‐transformed per‐cell green fluorescence (as 1+ *F*_*green*_, to allow presentation of zero‐count data) in the cyanobacterium *Synechococcus elongatus* (A–D) and the chlorophytes *Chlamydomonas_cf*. sp. (E–H), *Dunaliella tertiolecta* (I–L), and *Micractinium* sp. (M–P). Untreated and heat‐treated cultures were assayed without stains (first column), and stained with FDA, CMFDA, and FDA+CMFDA in the following columns. The vertical dashed lines in each panel are $F_{Threshold}^{Dead}$, as the 95th percentile of the distribution of per‐cell fluorescence intensity in the heat‐treated populations. The percentage of false negatives, untreated (live) cells with fluorescence lower than $F_{Threshold}^{Dead}$ is shown for each stain. The replicate shown was in each case the one (of 3 or 5, see text) with the median rate of false negatives with FDA+CMFDA.

**Figure S2.** Frequency distributions of log‐transformed per‐cell green fluorescence (as 1+ *F*_*green*_, to allow presentation of zero‐count data) in the chlorophytes *Nephroselmis pyriformis* (A–D), *Pyramimonas parkeae* (E–H), and *Scenedesmus obliquus* (I–L), and the euglenoid *Eutreptiella cf. gymnastica* (M–P). Untreated and heat‐treated cultures were assayed without stains (first column), and stained with FDA, CMFDA, and FDA+CMFDA in the following columns. The vertical dashed lines in each panel are $F_{Threshold}^{Dead}$, as the 95th percentile of the distribution of per‐cell fluorescence intensity in the heat‐treated populations. The percentage of false negatives, untreated (live) cells with fluorescence lower than $F_{Threshold}^{Dead}$ is shown for each stain. The replicate shown was in each case the one (of 3 or 5, see text) with the median rate of false negatives with FDA+CMFDA.

**Figure S3.** Frequency distributions of log‐transformed per‐cell green fluorescence (as 1+ *F*_*green*_, to allow presentation of zero‐count data) in the cryptophytes *Rhodomonas salina* (A–D) and *Storeatula* sp. (E–H), and the haptophytes *Chrysochromulina kappa* (I–L), and *Isochrysis galbana* (M–P). Untreated and heat‐treated cultures were assayed without stains (first column), and stained with FDA, CMFDA, and FDA+CMFDA in the following columns. The vertical dashed lines in each panel are $F_{Threshold}^{Dead}$, as the 95th percentile of the distribution of per‐cell fluorescence intensity in the heat‐treated populations. The percentage of false negatives, untreated (live) cells with fluorescence lower than $F_{Threshold}^{Dead}$ is shown for each stain. The replicate shown was in each case the one (of 3 or 5, see text) with the median rate of false negatives with FDA+CMFDA.

**Figure S4.** Frequency distributions of log‐transformed per‐cell green fluorescence (as 1+ *F*_*green*_, to allow presentation of zero‐count data) in the haptophytes *Phaeocystis globosa* (A–D) and *Prymnesium parvum* (E–H), and the diatoms *Amphiprora* sp. (I–L), and *Chaetoceros simplex* (M–P). Untreated and heat‐treated cultures were assayed without stains (first column), and stained with FDA, CMFDA, and FDA+CMFDA in the following columns. The vertical dashed lines in each panel are $F_{Threshold}^{Dead}$, as the 95th percentile of the distribution of per‐cell fluorescence intensity in the heat‐treated populations. The percentage of false negatives, untreated (live) cells with fluorescence lower than $F_{Threshold}^{Dead}$ is shown for each stain. The replicate shown was in each case the one (of 3 or 5, see text) with the median rate of false negatives with FDA+CMFDA.

**Figure S5.** Frequency distributions of log‐transformed per‐cell green fluorescence (as 1+ *F*_*green*_, to allow presentation of zero‐count data) in the raphidophyte *Heterosigma akashiwo* (A–D), the diatoms *Thalassiosira pseudonana* (E–H) and *Thalassiosira weissflogii* (I–L), and the dinoflagellate *Alexandrium andersoni* (M–P). Untreated and heat‐treated cultures were assayed without stains (first column), and stained with FDA, CMFDA, and FDA+CMFDA in the following columns. The vertical dashed lines in each panel are $F_{Threshold}^{Dead}$, as the 95th percentile of the distribution of per‐cell fluorescence intensity in the heat‐treated populations. The percentage of false negatives, untreated (live) cells with fluorescence lower than $F_{Threshold}^{Dead}$ is shown for each stain. The replicate shown was in each case the one (of 3 or 5, see text) with the median rate of false negatives with FDA+CMFDA.

**Figure S6.** Frequency distributions of log‐transformed per‐cell green fluorescence (as 1+ *F*_*green*_, to allow presentation of zero‐count data) in the dinoflagellates *Amphidinium carterae* (A–D), *Heterocapsa* sp. (E–H), *Prorocentrum triestinum* (I–L), and *Scrippsiella trochoidea* (M–P). Untreated and heat‐treated cultures were assayed without stains (first column), and stained with FDA, CMFDA, and FDA+CMFDA in the following columns. The vertical dashed lines in each panel are $F_{Threshold}^{Dead}$, as the 95th percentile of the distribution of per‐cell fluorescence intensity in the heat‐treated populations. The percentage of false negatives, untreated (live) cells with fluorescence lower than $F_{Threshold}^{Dead}$ is shown for each stain. The replicate shown was in each case the one (of 3 or 5, see text) with the median rate of false negatives with FDA+CMFDA.
