# Supplementary figures and images for: Classification of phytoplankton cells as live or dead using the vital stains fluorescein diacetate and 5‐chloromethylfluorescein diacetate
Source: J Phycol. 2016 Apr 28;52(4):572–89. doi: 10.1111/jpy.12415 (PMC5074294; doi:10.1111/jpy.12415)

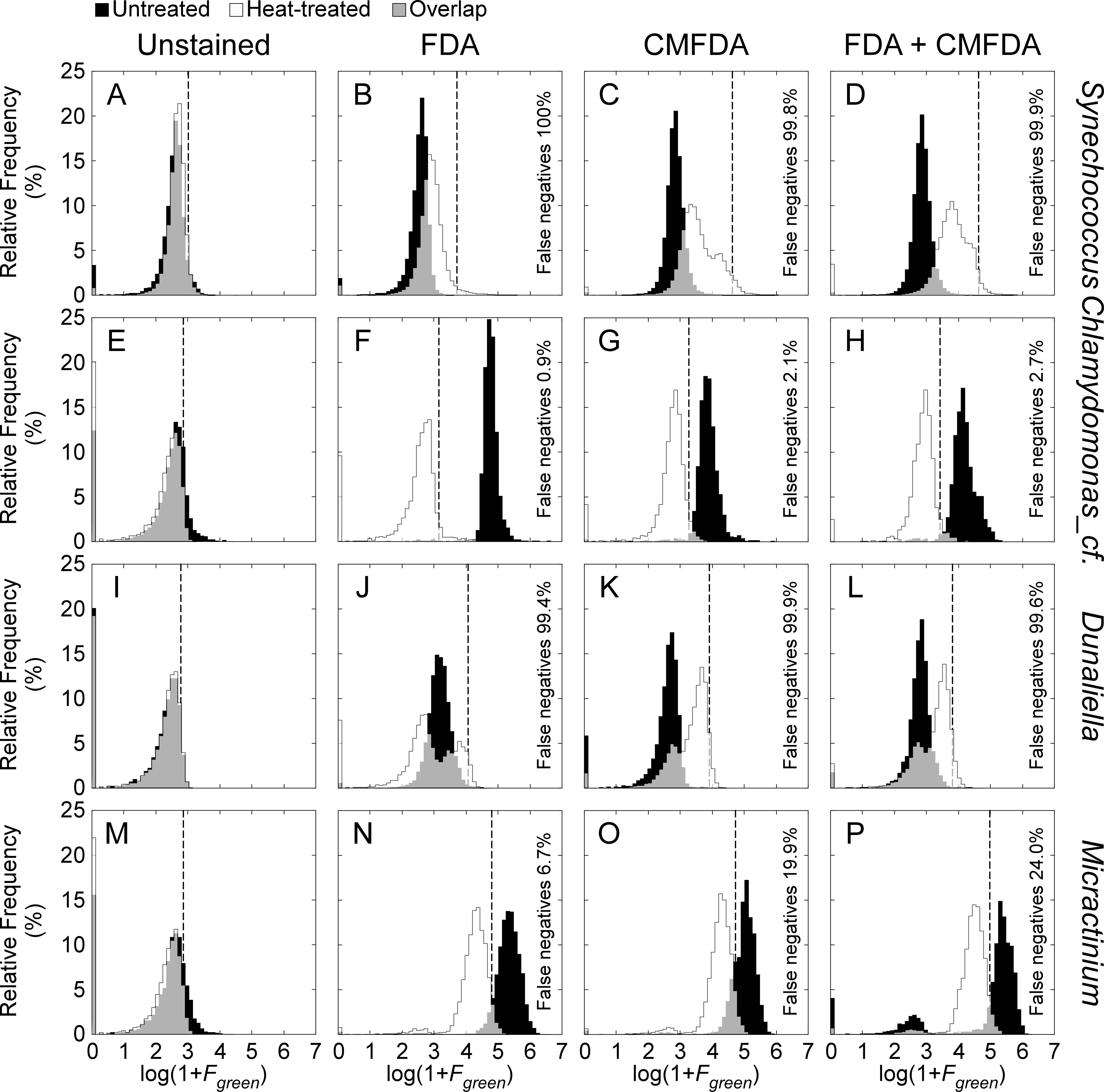

Supplement: Supplementary file 1 — Figure S1. Frequency distributions of log‐transformed per‐cell green fluorescence (as 1+ F green, to allow presentation of zero‐count data) in the cyanobacterium Synechococcus elongatus (A–D) and the chlorophytes Chlamydomonas_cf. sp. (E–H), Dunaliella tertiolecta (I–L), and Micractinium sp. (M–P). Untreated and heat‐treated cultures were assayed without stains (first column), and stained with FDA, CMFDA, and FDA+CMFDA in the following columns. The vertical dashed lines in each panel are FThresholdDead, as the 95th percentile of the distribution of per‐cell fluorescence intensity in the heat‐treated populations. The percentage of false negatives, untreated (live) cells with fluorescence lower than FThresholdDead is shown for each stain. The replicate shown was in each case the one (of 3 or 5, see text) with the median rate of false negatives with FDA+CMFDA. [file JPY-52-572-s001.gif]

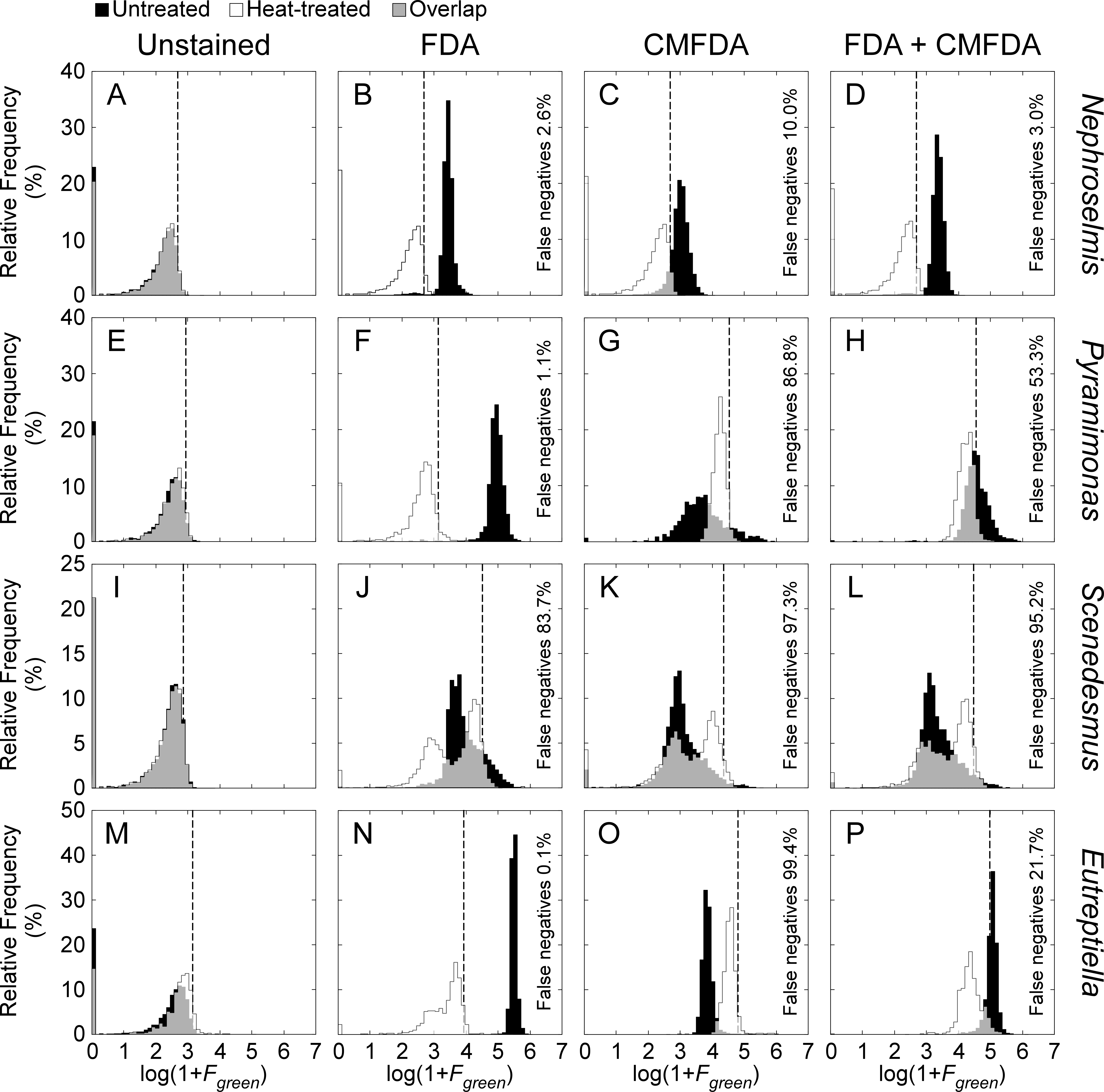

Supplement: Supplementary file 2 — Figure S2. Frequency distributions of log‐transformed per‐cell green fluorescence (as 1+ F green, to allow presentation of zero‐count data) in the chlorophytes Nephroselmis pyriformis (A–D), Pyramimonas parkeae (E–H), and Scenedesmus obliquus (I–L), and the euglenoid Eutreptiella cf. gymnastica (M–P). Untreated and heat‐treated cultures were assayed without stains (first column), and stained with FDA, CMFDA, and FDA+CMFDA in the following columns. The vertical dashed lines in each panel are FThresholdDead, as the 95th percentile of the distribution of per‐cell fluorescence intensity in the heat‐treated populations. The percentage of false negatives, untreated (live) cells with fluorescence lower than FThresholdDead is shown for each stain. The replicate shown was in each case the one (of 3 or 5, see text) with the median rate of false negatives with FDA+CMFDA. [file JPY-52-572-s002.gif]

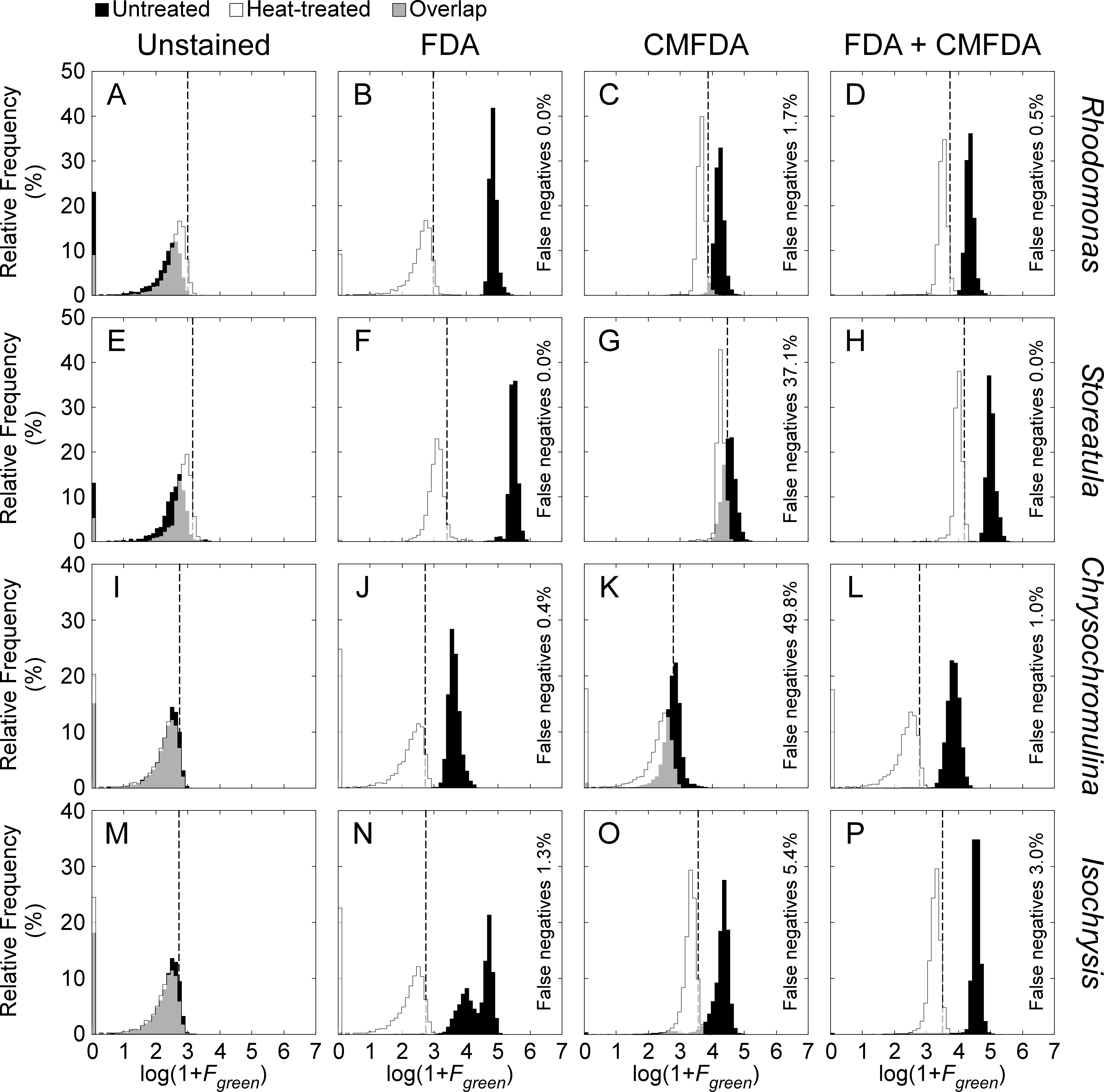

Supplement: Supplementary file 3 — Figure S3. Frequency distributions of log‐transformed per‐cell green fluorescence (as 1+ F green, to allow presentation of zero‐count data) in the cryptophytes Rhodomonas salina (A–D) and Storeatula sp. (E–H), and the haptophytes Chrysochromulina kappa (I–L), and Isochrysis galbana (M–P). Untreated and heat‐treated cultures were assayed without stains (first column), and stained with FDA, CMFDA, and FDA+CMFDA in the following columns. The vertical dashed lines in each panel are FThresholdDead, as the 95th percentile of the distribution of per‐cell fluorescence intensity in the heat‐treated populations. The percentage of false negatives, untreated (live) cells with fluorescence lower than FThresholdDead is shown for each stain. The replicate shown was in each case the one (of 3 or 5, see text) with the median rate of false negatives with FDA+CMFDA. [file JPY-52-572-s003.gif]

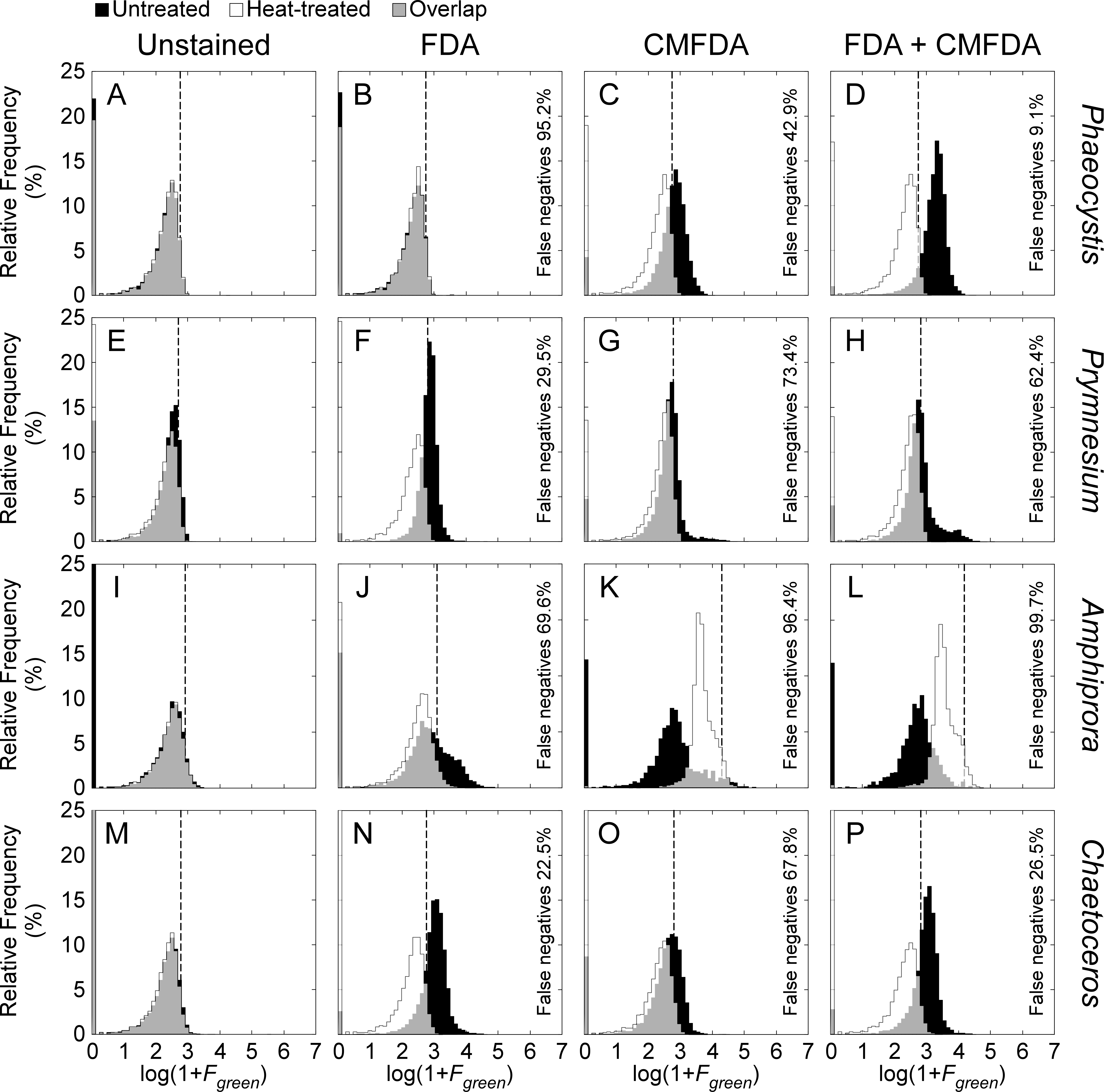

Supplement: Supplementary file 4 — Figure S4. Frequency distributions of log‐transformed per‐cell green fluorescence (as 1+ F green, to allow presentation of zero‐count data) in the haptophytes Phaeocystis globosa (A–D) and Prymnesium parvum (E–H), and the diatoms Amphiprora sp. (I–L), and Chaetoceros simplex (M–P). Untreated and heat‐treated cultures were assayed without stains (first column), and stained with FDA, CMFDA, and FDA+CMFDA in the following columns. The vertical dashed lines in each panel are FThresholdDead, as the 95th percentile of the distribution of per‐cell fluorescence intensity in the heat‐treated populations. The percentage of false negatives, untreated (live) cells with fluorescence lower than FThresholdDead is shown for each stain. The replicate shown was in each case the one (of 3 or 5, see text) with the median rate of false negatives with FDA+CMFDA. [file JPY-52-572-s004.gif]

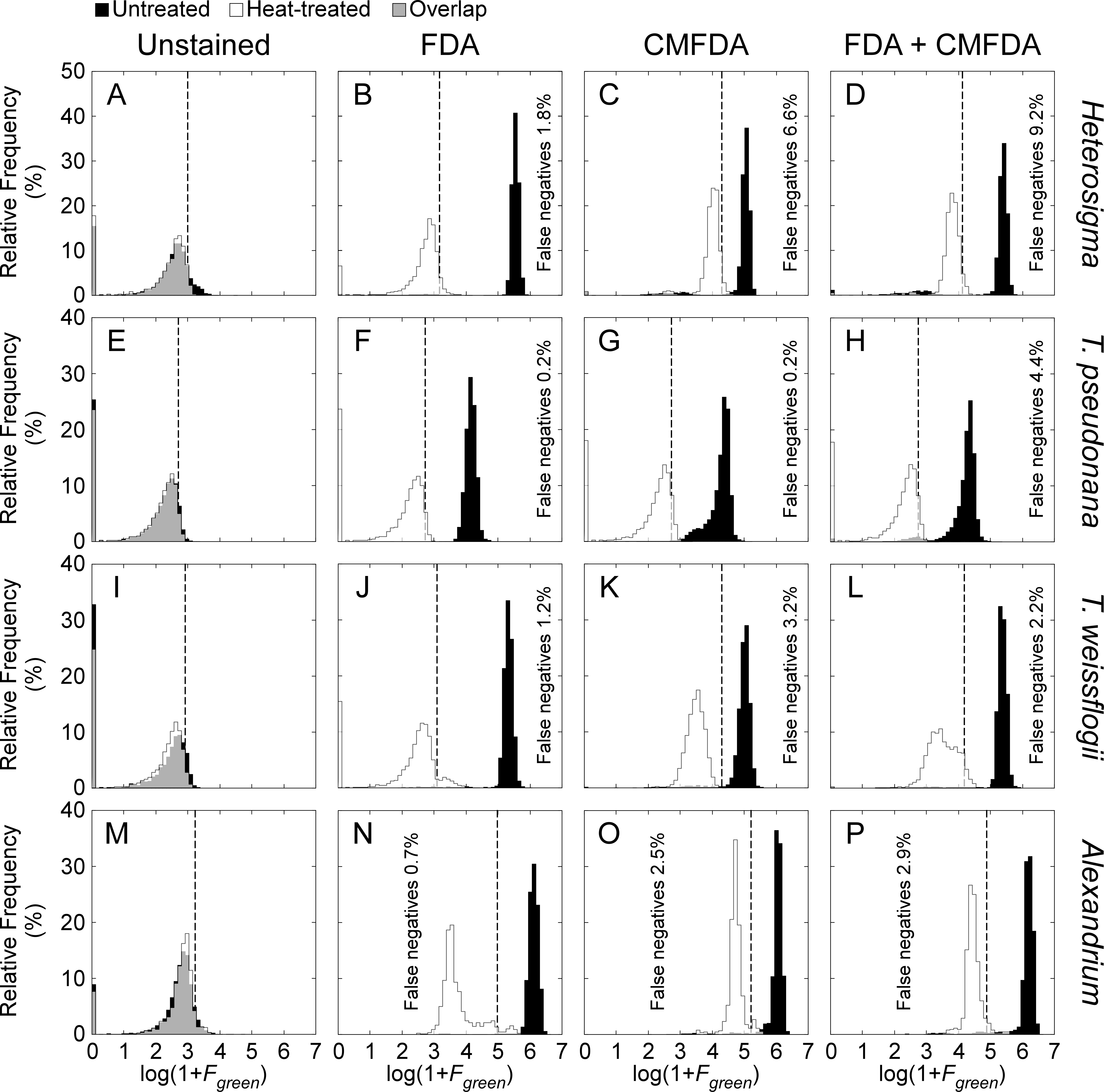

Supplement: Supplementary file 5 — Figure S5. Frequency distributions of log‐transformed per‐cell green fluorescence (as 1+ F green, to allow presentation of zero‐count data) in the raphidophyte Heterosigma akashiwo (A–D), the diatoms Thalassiosira pseudonana (E–H) and Thalassiosira weissflogii (I–L), and the dinoflagellate Alexandrium andersoni (M–P). Untreated and heat‐treated cultures were assayed without stains (first column), and stained with FDA, CMFDA, and FDA+CMFDA in the following columns. The vertical dashed lines in each panel are FThresholdDead, as the 95th percentile of the distribution of per‐cell fluorescence intensity in the heat‐treated populations. The percentage of false negatives, untreated (live) cells with fluorescence lower than FThresholdDead is shown for each stain. The replicate shown was in each case the one (of 3 or 5, see text) with the median rate of false negatives with FDA+CMFDA. [file JPY-52-572-s005.gif]

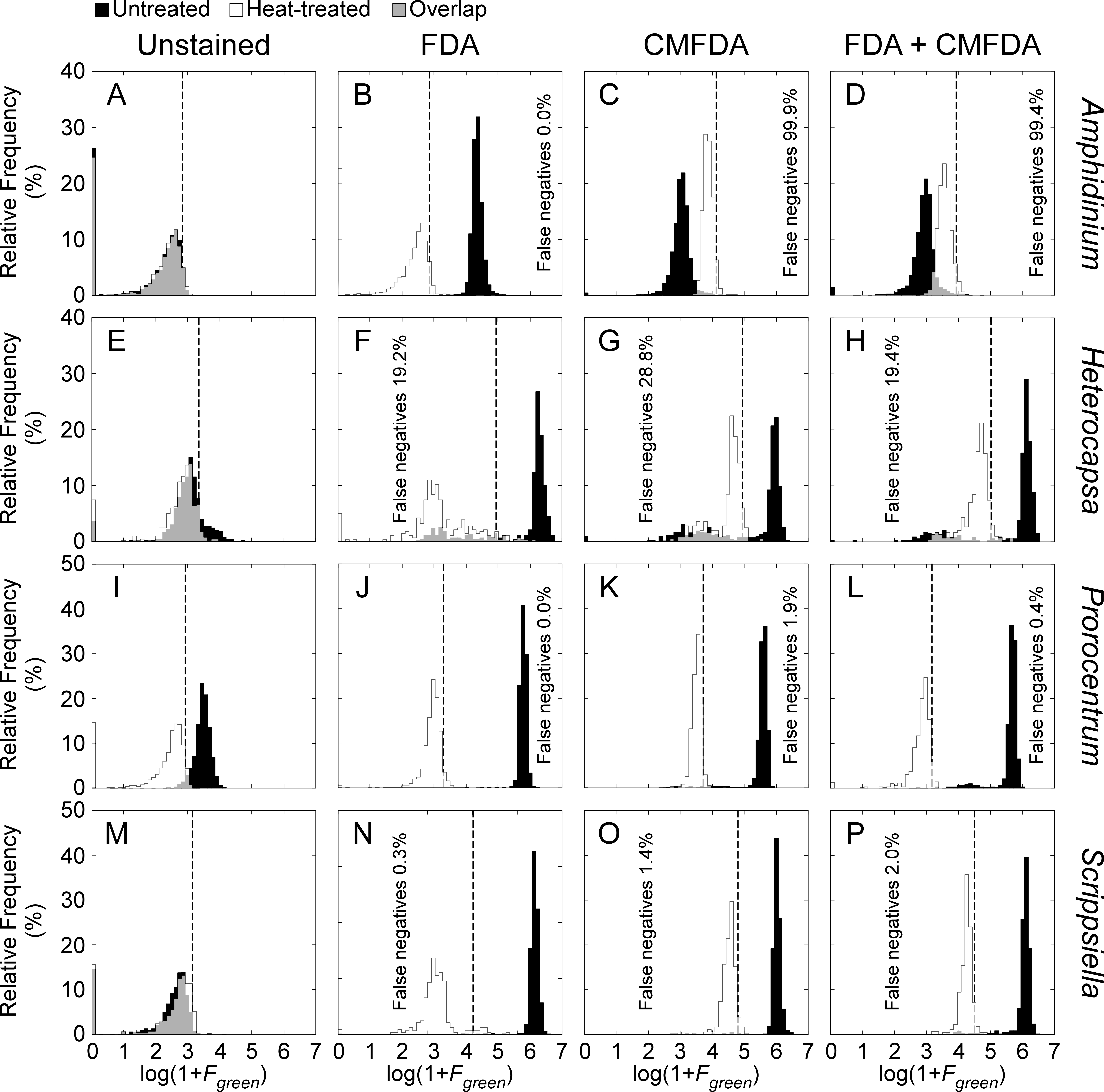

Supplement: Supplementary file 6 — Figure S6. Frequency distributions of log‐transformed per‐cell green fluorescence (as 1+ F green, to allow presentation of zero‐count data) in the dinoflagellates Amphidinium carterae (A–D), Heterocapsa sp. (E–H), Prorocentrum triestinum (I–L), and Scrippsiella trochoidea (M–P). Untreated and heat‐treated cultures were assayed without stains (first column), and stained with FDA, CMFDA, and FDA+CMFDA in the following columns. The vertical dashed lines in each panel are FThresholdDead, as the 95th percentile of the distribution of per‐cell fluorescence intensity in the heat‐treated populations. The percentage of false negatives, untreated (live) cells with fluorescence lower than FThresholdDead is shown for each stain. The replicate shown was in each case the one (of 3 or 5, see text) with the median rate of false negatives with FDA+CMFDA. [file JPY-52-572-s006.gif]
